# NLRP6 negatively regulates pulmonary host defense in Gram-positive bacterial infection through modulating neutrophil recruitment and function

**DOI:** 10.1371/journal.ppat.1007308

**Published:** 2018-09-24

**Authors:** Laxman Ghimire, Sagar Paudel, Liliang Jin, Pankaj Baral, Shanshan Cai, Samithamby Jeyaseelan

**Affiliations:** 1 Lung Biology Laboratory, Department of Pathobiological Sciences, School of Veterinary Medicine, Louisiana State University (LSU), Baton Rouge, LA, United States of America; 2 Section of Pulmonary and Critical Care, Department of Medicine, LSU Health Science Center, New Orleans, LA, United States of America; University of Toronto, CANADA

## Abstract

Gram-positive bacteria, including *Staphylococcus aureus* are endemic in the U.S., which cause life-threatening necrotizing pneumonia. Neutrophils are known to be critical for clearance of *S*. *aureus* infection from the lungs and extrapulmonary organs. Therefore, we investigated whether the NLRP6 inflammasome regulates neutrophil-dependent host immunity during pulmonary *S*. *aureus* infection. Unlike their wild-type (WT) counterparts, NLRP6 knockout (KO) mice were protected against pulmonary *S*. *aureus* infection as evidenced by their higher survival rate and lower bacterial burden in the lungs and extrapulmonary organs. In addition, NLRP6 KO mice displayed increased neutrophil recruitment following infection, and when neutrophils were depleted the protective effect was lost. Furthermore, neutrophils from the KO mice demonstrated enhanced intracellular bacterial killing and increased NADPH oxidase-dependent ROS production. Intriguingly, we found higher NK cell-mediated IFN-γ production in KO mouse lungs, and treatment with IFN-γ was found to enhance the bactericidal ability of WT and KO neutrophils. The NLRP6 KO mice also displayed decreased pyroptosis and necroptosis in the lungs following infection. Blocking of pyroptosis and necroptosis in WT mice resulted in increased survival, reduced bacterial burden in the lungs, and attenuated cytokine production. Taken together, these novel findings show that NLRP6 serves as a negative regulator of neutrophil-mediated host defense during Gram-positive bacterial infection in the lungs through regulating both neutrophil influx and function. These results also suggest that blocking NLRP6 to augment neutrophil-associated bacterial clearance should be considered as a potential therapeutic intervention strategy for treatment of *S*. *aureus* pneumonia.

## Author summary

Gram-positive bacteria, including *Staphylococcus aureus* remain a major cause of acute pneumonia worldwide. Due to emergence of multidrug-resistant strains, alternative strategies for treatment of *S*. *aureus* pneumonia are needed. To this end, it may be possible to harness host defenses to eradicate the infection instead of directly targeting the bacteria. Neutrophils are a crucial innate immune cell type and serve as a first line of defense against bacterial lung infection. NLRP6 is a recently identified member of Nod-like receptor family. Nonetheless, the molecular and cellular immunological mechanisms by which the NLRP6 regulates neutrophil-mediated host immunity during acute *S*. *aureus* pneumonia remain elusive. We found that NLRP6 gene-deficient/knockout (KO) mice demonstrate increased survival and lower bacterial burden in the lungs along with enhanced neutrophil recruitment during acute *S*. *aureus* pneumonia. Moreover, neutrophils from NLRP6 KO mice showed increased bactericidal ability compared to those from controls. Similarly, NLRP6 KO mice demonstrated decreased cell death through pyroptosis and necroptosis following infection. Blocking of these cell death mechanisms in WT mice resulted in increased survival and decreased bacterial burden in the lungs following infection. Therefore, our study provides novel insights into the novel mechanisms mediated by NLRP6, which serves as a negative regulator of neutrophil-mediated host defense during Gram-positive pneumonia.

## Introduction

Acute pneumonia is a leading cause of childhood mortality (<5 years of age) accounting for the death of 920,136 children annually [1], and methicillin-resistant *Staphylococcus aureus* (MRSA) has been implicated in severe life-threatening infections, including necrotizing pneumonia and sepsis [2]. In addition, *S*. *aureus* infection is also one of the major causes of secondary pneumonia following influenza infection [3]. Furthermore, *S*. *aureus* has developed resistance to multiple antibiotics and effective treatment strategies against this bacterium are limited [2, 4]. Therefore, *S*. *aureus* is a serious threat to human health and novel therapeutic strategies are needed.

The lung pathology induced by *S*. *aureus* is attributed to virulence factors, the intense inflammatory response, and evasion of host defense mechanisms, including neutrophil-mediated ROS-dependent bacterial killing [5, 6]. Regarding innate immune responses, nucleotide-binding oligomerization domain-like receptor (NLR) pyrin domain-6 (NLRP6) is a recently identified NLR present in the cytosol of innate immune cells [7]. Co-transfection of plasmids containing NLRP6 and apoptosis-associated speck-like protein containing card (ASC) in 293T cells led to activation of NF-kB and in COS-7L cells to a synergistic increase in caspase-1 activation and IL-1β secretion [8]. Under homeostatic conditions Levy *et al* have demonstrated that NLRP6 co-localizes with ASC and caspase-1 to form a complex resulting in IL-18 secretion from intestinal epithelial cells, which is essential to prevent development of dysbiosis [9]. Together these results suggest that NLRP6 can co-localize with ASC for inflammasome formation and activation. However, the mechanisms of NLRP6 inflammasome activation and its role in host defense specifically during pulmonary microbial infection has not been explored. Moreover, it remains unknown whether the NLRP6 inflammasome is activated by microbial infections and whether NLRP6 co-localizes with ASC and caspase-1 during such infections to induce pyroptosis.

In a mouse model of systemic infection, Anand *et a*l [7] found that the NLRP6 negatively regulates host defense against *Listeria* and *Salmonella* infections as NLRP6 KO mice showed higher survival, decreased bacterial burden, and attenuated pathology compared to WT mice. In contrast, a study reported by Wlodarska *et al* [10] revealed a positive regulatory role of the NLRP6 inflammasome on immune function during enteric infection with *Citrobacter rodentium*. In this investigation, NLRP6 KO mice were shown to have an increased *C*. *rodentium* burden in the intestine, which correlated with extensive mucosal damage in the KO mice compared with WT controls. However, these results cannot be extrapolated to other bacterial infections in different organs, such as the lung, because Gram-positive bacteria have unique virulence factors and immune evasion strategies and the route of administration of bacteria also dictates host responses. Thus, the role of the NLRP6 inflammasome in pulmonary immunity against Gram-positive infections remains unclear. To this end, we have used NLRP6 KO mice to demonstrate how *S*. *aureus* exploits the NLRP6 inflammasome to dampen neutrophil function and enhance pyroptosis and necroptosis to increase mortality during acute bacterial pneumonia. Our results show NLRP6 as a potential therapeutic target for treatment of *S*. *aureus*-infected pneumonic patients.

## Results

### NLRP6 expression is increased in the lungs of pneumonic patients and *S*. *aureus*-infected mice

**Fig 1:**
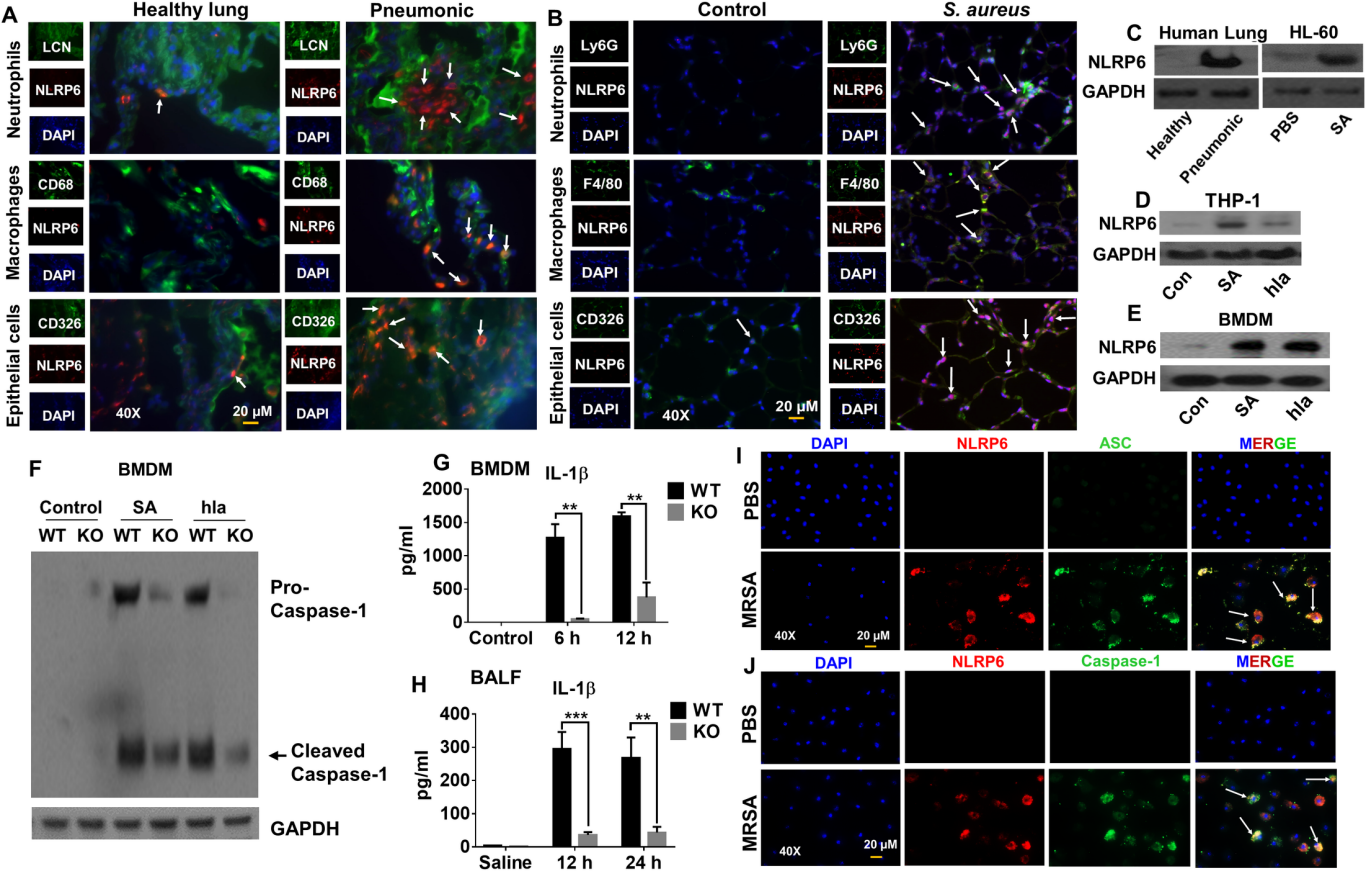
Upregulation and activation of NLRP6. **(A)** Lungs tissue sections from healthy controls and pneumonic patients were stained with antibodies against neutrophils (lipocalin-2^+^), macrophages (CD68^+^), and epithelial (CD326^+^) cells. NLRP6 is shown in red, neutrophils, macrophages, and epithelial cell markers are in green, and DAPI is blue. NLRP6-positive cells are indicated by white arrowheads. **(B)** Immunofluorescence microscopy was performed on lung sections from healthy controls and *S*. *aureus*-infected mice to assess expression of NLRP6. Neutrophils (Ly6G^+^), macrophages (F4/80^+^), and epithelial cells (CD 326^+^) are stained green and NLRP6 is red. NLRP6-positive cells are indicated by white arrowheads. **(C)** NLRP6 expression was analyzed by western blot in lysates of human healthy control tissue, pneumonic lung tissue, and HL-60 (human neutrophil-like) cells infected with *S*. *aureus* (MOI10) for 4 hours. **(D)** THP-1 (human monocytic) cells were infected with *S*. *aureus* (MOI 20) or purified α-hemolysin (hla) for 8 hours and expression of NLRP6 was assessed by western blotting. **(E, F)** Bone marrow-derived macrophages (BMDMs) were infected with *S*. *aureus* (MOI: 20) or hla from *S*. *aureus* (50 μg/ml) for 8 hours **(E)**. Expression of NLRP6 protein was analyzed by immunoblotting. **(F)** Caspase-1 processing by the NLRP6 inflammasome in response to *S*. *aureus* or hla in BMDMs. **(G)** IL-1β secretion by WT and NLRP6 KO BMDMs following *S*. *aureus* infection. **(H)** IL-1β secretion by WT and NLRP6 KO mice after *S*. *aureus* (5 X 10^7^ CFU/mouse) infection (N = 6-8/group). **(I)** BMDMs from WT mice were infected with *S*. *aureus* (MOI: 50) and stained for NLRP6 (red) and ASC (green). The white arrowheads demonstrate co-localization of NRP6 and ASC. **(J)** BMDMs from WT mice were infected with *S*. *aureus* (MOI: 50) and stained for NLRP6 (red) and caspase-1 (green). The white arrowheads show co-localization of NRP6 and caspase-1. The graphs show the mean ± SEM of three independent experiments. The images shown are the representative of five different fields from three independent experiments. Magnification: 40X. SA: *S*. *aureus*, hla: α-hemolysin. *, p<0.05, **, p<0.01, and *** p<0.001.

To determine whether NLRP6 is upregulated in the lungs of pneumonic patients, we stained human pneumonic and normal lung tissue sections with anti-NLRP6 antibody and found that NLPR6 was upregulated in key innate immune cells in the lungs, such as neutrophils (lipocalin-2^+^), macrophages (CD68^+^), and epithelial cells (CD326^+^) (Fig 1A). Next, we determined if NLRP6 expression is upregulated in *S*. *aureus*-induced pneumonia in mice. In line with the results seen in human pneumonic lung sections, NLRP6 was upregulated in neutrophils (Ly6G^+^), macrophages (F4/80^+^), and epithelial cells (CD 326^+^) of mouse lungs following *S*. *aureus* infection (Fig 1B). Consistent with the immunofluorescence results, the expression of NLRP6 was also increased in lung lysates of human pneumonic patients, *S*. *aureus*-infected human cell lines (THP-1 and HL-60), and mouse bone marrow-derived macrophages (BMDMs) (Fig 1C–1E).

To investigate whether NLRP6 triggers inflammasome activation during *S*. *aureus* infection, we infected BMDMs from WT and NLRP6 KO (KO) mice with *S*. *aureus* (MOI: 20) and measured the extent of caspase-1 activation at 8 hours post-infection. Both cleaved caspase-1 (p-20) in macrophage lysates and IL-1β levels in culture supernatants following infection were attenuated in the NLRP6 KO samples compared to the WT control (Fig 1F and 1G) indicating the activation of the NLRP6 inflammasome by *S*. *aureus*. Similarly, IL-18 was also reduced in NLRP6 KO BMDMs after *S*. *aureus* infection (S1A Fig). Since IL-1β was sharply reduced in the KO BMDMs, we determined if NLRP3 inflammasome is still intact in these BMDMs. IL-1β production in NLRP6 KO BMDMs after treatment with specific NLRP3 agonists (ATP and Nigericin) was comparable with that of WT BMDMs suggesting that NLRP3 is indeed intact in NLRP6 KO macrophages (S1B Fig). *S*. *aureus* has several virulence components, such as α-hemolysin (hla), clumping factor B, β toxin, phenol-soluble modulins, and panton-valentine leukocidins [5] that could potentially activate the NLRP6 inflammasome. Because hla has been reported to activate the NLRP3 inflammasome in human and mouse monocytic cells under similar conditions [11, 12], we investigated whether hla can also activate the NLRP6 inflammasome in BMDMs and found this to be the case (Fig 1E and 1F). Consistent with the *in vitro* results, NLRP6 KO mice showed lower levels of IL- 1β in bronchoalveolar lavage fluid (BALF) after infection with *S*. *aureus*, providing evidence of NLRP6 inflammasome activation *in vivo* (Fig 1H). However, there was no difference observed in the levels of IL-18 between WT and the KO mice (S1C Fig). ASC is known to be an integral part of NLRP3 inflammasome signaling although it is dispensable for NLRC4 inflammasome activation [13]. We infected BMDMs with *S*. *aureus* and performed immunofluorescence assay to observe whether NLRP6 co-localizes with ASC and caspase-1. We found that NLRP6 co-localized with ASC and caspase-1 during *S*. *aureus* infection (Fig 1I and 1J and S1D Fig).

### NLRP6 deficiency confers host protection during *S*. *aureus*-induced pneumonia

**Fig 2:**
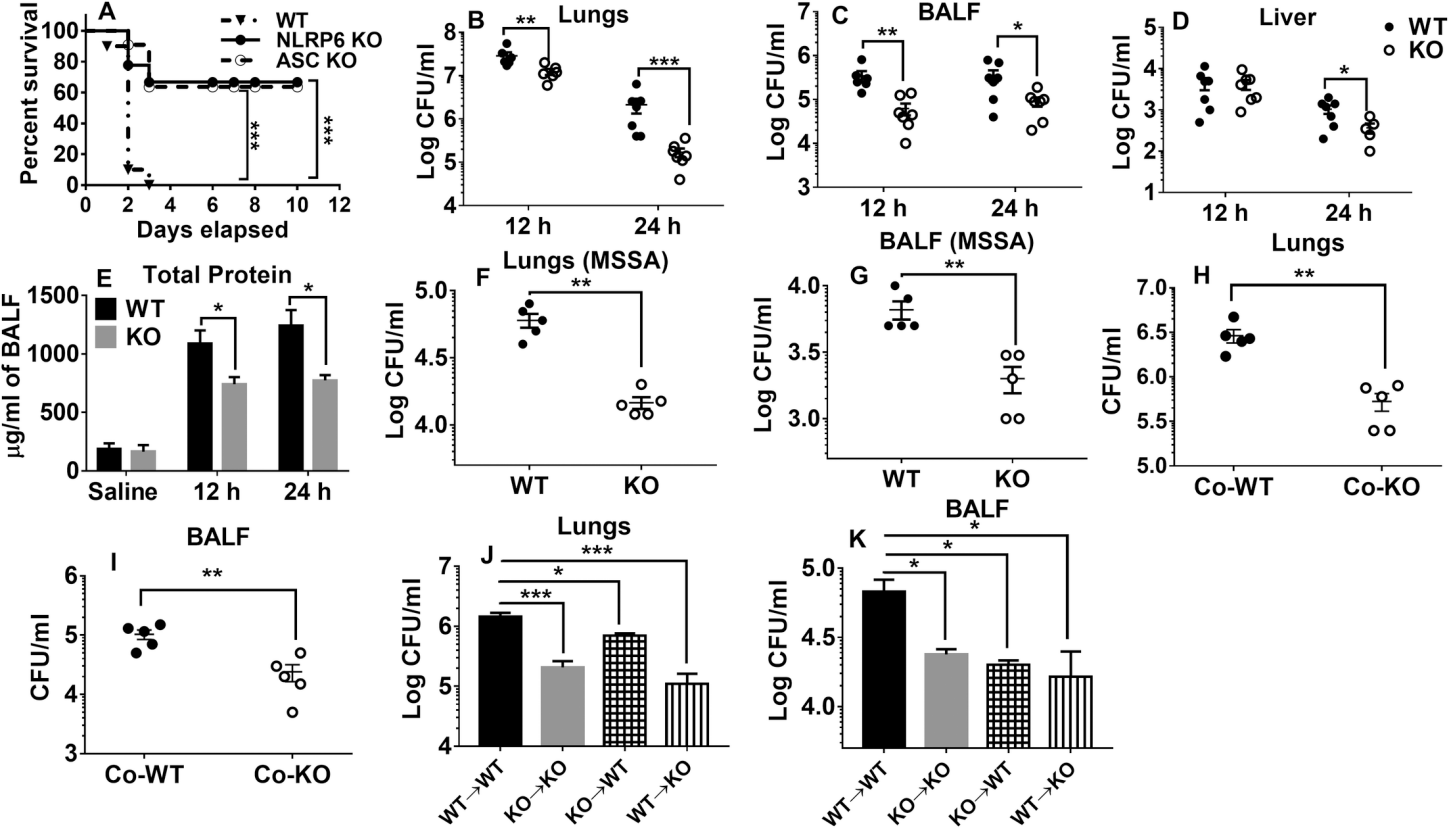
NLRP6^-/-^ mice are resistant to pulmonary *S*. *aureus* infection. **(A)** WT, NLRP6 KO, and ASC KO mice (N = 13-15/group) were infected intratracheally with a lethal inoculum of *S*. *aureus* (USA 300) (2X10^8^ CFU/mouse) and survival was then observed for 10 days. WT and NLRP6 KO mice (N = 5-8/group) were infected intratracheally with a sublethal inoculum of *S*. *aureus* (5 X 10^7^ CFU/mouse) and then were euthanized 12 or 24 h post-infection to quantitate the bacterial burden in **(B)** lungs, **(C)** BALF, and **(D)** liver. Total protein in BALF was measured **(E).** WT and KO mice (N = 6-8/group) were infected intratracheally with 5X10^7^ CFU/mouse of *S*. *aureus* (MSSA strain). Twenty-four-hours post-infection, the bacterial burden was measured in lungs **(F)** and BALF **(G)**. WT and KO mice (N = 5/group) were co-housed for 4 weeks and infected with a sublethal dose of *S*. *aureus*. Mice were euthanized at 24 hours post-infection to estimate bacterial burden in the lungs **(H)** and BALF **(I)**. Bone marrow chimeras were generated as described in the methods (N = 6-8/group) and then were infected with a sublethal dose of *S*. *aureus*. At 24-hours post-infection, mice were euthanized to estimate bacterial burden in the lungs **(J)** and BALF **(K).** *, p<0.05, **, p<0.01, and *** p<0.001.

To assess the role of the NLRP6 inflammasome in pulmonary host defense against *S*. *aureus*, we infected WT, ASC KO, and NLRP6 KO mice intratracheally with a lethal dose of *S*. *aureus* (USA 300) (2X10^8^ CFUs per mouse) and observed the survival patterns for 10 days. Although all WT mice died within 3 days, 70% of NLRP6 KO mice survived longer than 10-days post-infection (Fig 2A). Furthermore, ASC KO mice displayed a survival pattern similar to that of the NLRP6 KO mice (Fig 2A). To determine whether the difference in survival is due to differences in the bacterial burden in various organs, we measured the bacterial burden in the lung, BALF, and extra-pulmonary organs after infecting mice with a sub-lethal inoculum (5X10^7^CFU) of *S*. *aureus*. As compared to NLRP6 KO mice, WT mice had higher bacterial burdens in the lungs, BALF, and liver at both 12 and 24-hours post-infection (Fig 2B–2D). Accordingly, the total protein in the BALF, which is a measure of pulmonary leakage, was higher in WT mice compared to their NLPR6 KO counterparts (Fig 2E).

To determine whether the detrimental effects of NLRP6 are bacterial strain specific, we infected WT and KO mice with a methicillin-susceptible *Staphylococcus aureus* strain (MSSA, Newman strain) and measured the bacterial burden in lungs and BALF at 24-hours post-infection. Consistent with the results seen with the USA 300 strain, the bacterial burden of the methicillin-sensitive strain was also higher in the lungs and BALF of WT mice compared to that of KO mice (Fig 2F and 2G). NLRP6 inflammasome has been implicated in regulating intestinal microbiota [9, 14]. To determine if observed difference in the phenotype between WT and NLRP6 KO mice is due to difference in gut microbiota, we co-housed WT and NLRP6 KO mice for 4 weeks and infected them with *S*. *aureus*. As observed with non-co-housed mice, co-housed NLRP6 KO mice had significantly less bacterial burden in the lungs and BALF as compared to co-housed WT mice (Fig 2H and 2I). These data suggest that NLRP6 regulates pulmonary *S*. *aureus* infection independent of microbiota composition.

### Both hematopoietic and non-hematopoietic cells contribute to host susceptibility

Since we found upregulation of NLRP6 in both hematopoietic (neutrophils and macrophages) and non-hematopoietic cells (epithelial cells) (Fig 1B), we sought to determine if NLRP6 from each of these compartments is detrimental to bacterial clearance of *S*. *aureus*-induced pneumonia. Using bone marrow chimeric mice, we found that WT mice that received KO bone marrow (KO→WT) had lower bacterial burdens in the lungs and BALF than WT mice that received WT bone marrow (WT→WT) (Fig 2J and 2K). However, KO mice that received WT bone marrow (WT→KO) showed no increase in bacterial burden in the lungs and BALF compared to KO mice that received KO bone marrow (KO→KO) (Fig 2J and 2K). Together, these results suggest that NLRP6 derived from both hematopoietic and non-hematopoietic compartments is detrimental for bacterial clearance during *S*. *aureus* pneumonia.

### Enhanced host protection in NLRP6 KO mice is dependent on neutrophils

**Fig 3:**
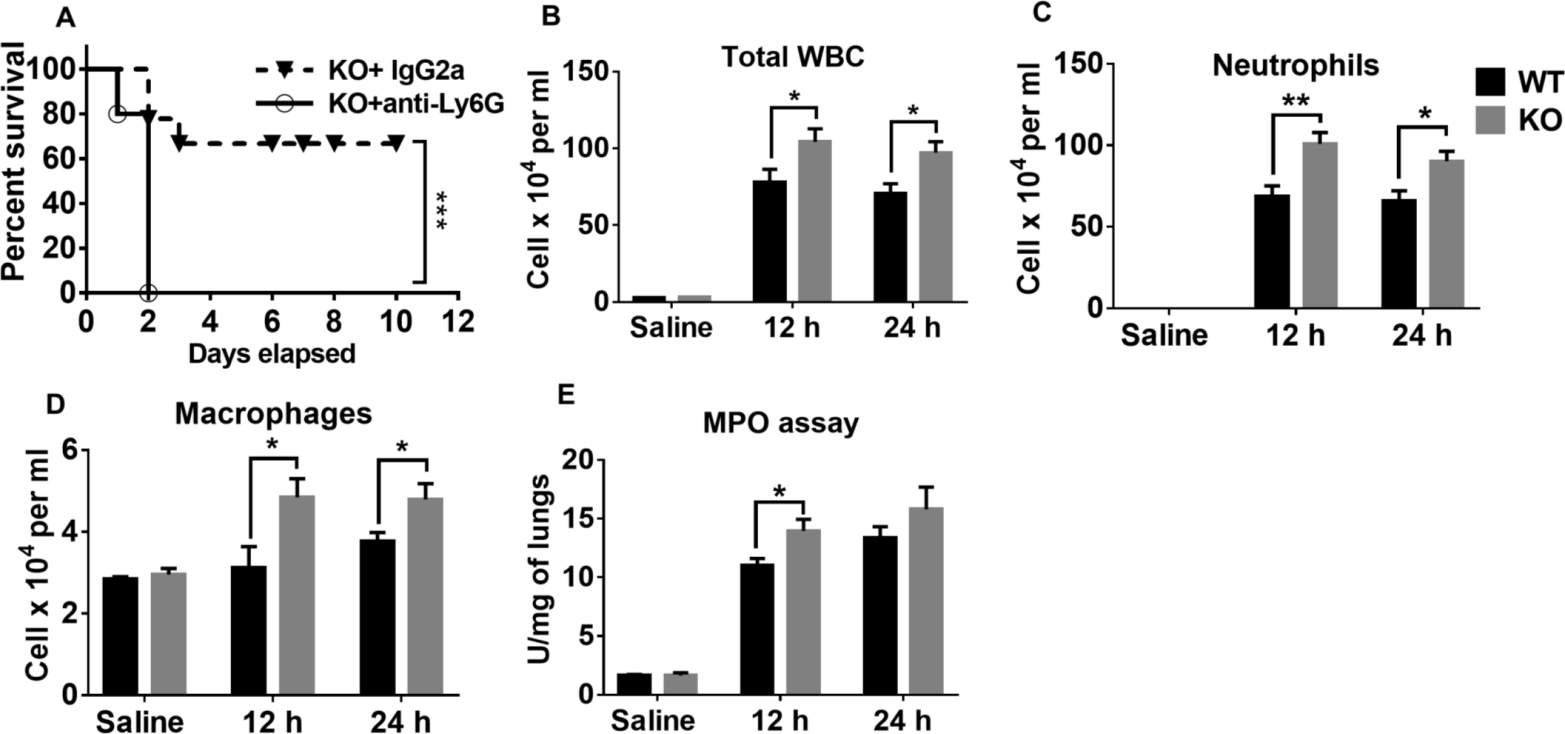
Neutrophils confer host protection in NLRP6 KO mice. **(A)** NLRP6 KO mice (N = 10-12/group) were treated either with anti-Ly6G antibody or IgG2a, 24 and 2 hours prior to infection with a lethal dose of *S*. *aureus* (USA300). Survival was monitored for 10 days. NLRP6 KO mice (N = 5-8/group) were infected intratracheally with a sublethal inoculum of *S*. *aureus* (5 X 10^7^ CFU/mouse). Mice were euthanized 12- or 24-hours post-infection to estimate total leukocytes **(B)**, neutrophils **(C)**, and macrophages **(D)** in the BALF. **(E)** Myeloperoxidase assay was performed in the lungs. *, p<0.05, **, p<0.01, and *** p<0.001.

Neutrophils have been shown to be essential for containing pulmonary *Staphylococcal* infections [15, 16]. To determine if neutrophils confer augmented host protection in NLRP6 KO mice, we depleted neutrophils in the KO mice prior to infection with a lethal dose of *S*. *aureus* and observed survival. We found that depletion of neutrophils reversed the survival benefit observed in the NLRP6 KO mice suggesting that protection is neutrophil-dependent (Fig 3A). Since neutrophils are essential for survival, we investigated if disruption of NLRP6 affects recruitment of neutrophils into alveolar spaces during *S*. *aureus* pneumonia. Compared to WT mice, KO mice had more leukocytes, including neutrophils, and macrophages recruited into alveolar spaces (Fig 3B–3D). Furthermore, to measure neutrophil accumulation in the lung parenchyma, we performed a myeloperoxidase (MPO) assay and found that KO mice had more MPO activity than WT mice (Fig 3E).

### Deletion of NLRP6 enhances bacterial killing of neutrophils through increased IFN-γ and ROS production

**Fig 4:**
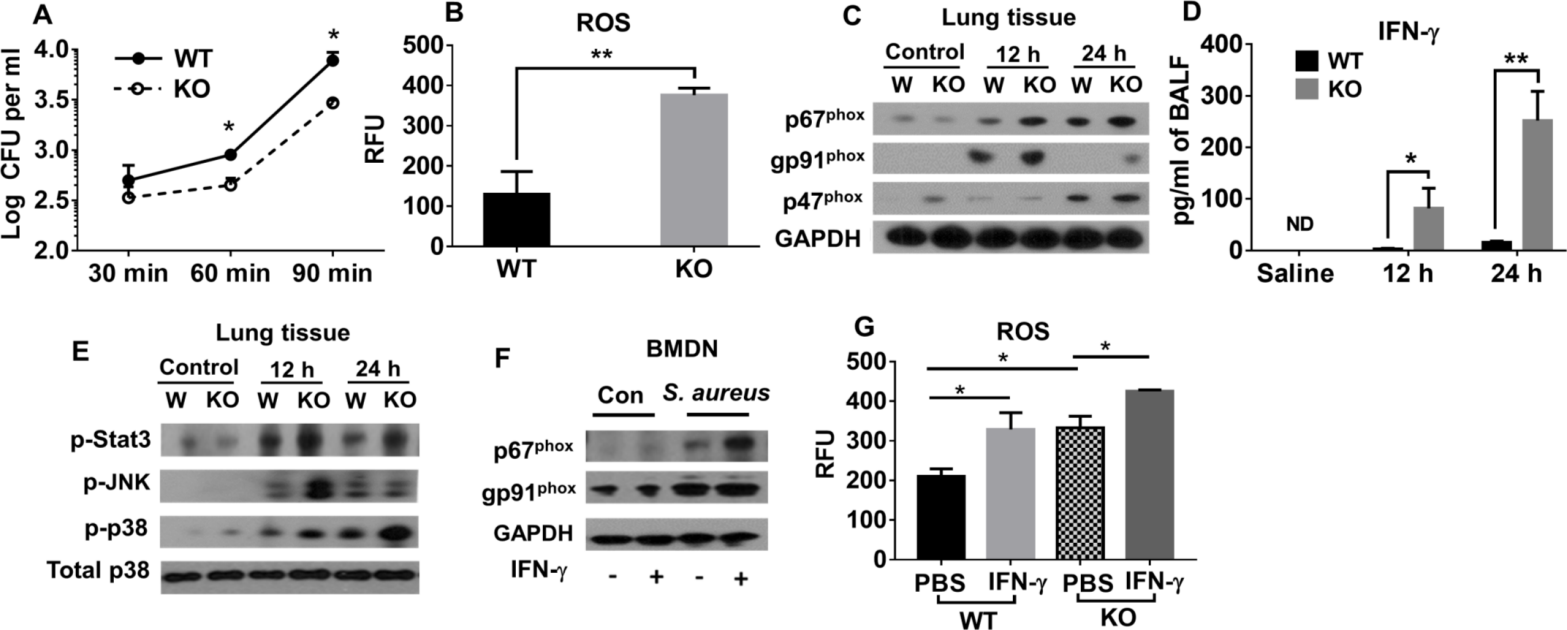
NLRP6^-/-^ neutrophils exhibit enhanced intracellular killing through increased IFN-γ and NADPH oxidase-dependent ROS production. **(A)** BMDNs from WT and KO mice were infected with *S*. *aureus* (MOI 10) and the intracellular killing ability of neutrophils was measured at the indicated time points. **(B)** ROS production by BMDNs from WT and KO mice were compared after infection with *S*. *aureus* (MOI 1). **(C)** WT and KO mice (N = 6-8/group) were infected with a sublethal dose of *S*. *aureus* (5 X 10^7^ CFU/mouse). At designated time points, lungs were collected and processed for immunoblotting to compare the expression of NADPH oxidase enzyme components between WT and KO mice. **(D)** IFN-γ in BALF obtained from mice in **3C** was measured. **(E)** Lungs obtained from *S*. *aureus*-infected WT and KO mice were homogenized and expressions of phospho-38, phospho-JNK, and phospho-STAT3 were measured through immunoblotting. **(F)** BMDNs from WT and KO mice were isolated and pre-treated with either IFN-γ or PBS for 30 minutes before infection with *S*. *aureus* (MOI 10). Two-hours post-infection, WT neutrophils were lysed and immunoblotted to measure the expression of NADPH oxidase enzyme components. **(G)** ROS production by neutrophils in **3F** were measured as described in the methods. All figures are representative of three independent experiments. ROS: reactive oxygen species, RFU: relative florescence unit. WT: Wild-type, KO: NLRP6 KO. *, p<0.05, **, p<0.01, and *** p<0.001.

Since NLRP6 was found to be upregulated in neutrophils and macrophages in the lungs, we wanted to know whether deletion of NLRP6 affects the function of these cells. To this end, we compared the intracellular killing ability of bone marrow derived-neutrophils (BMDNs) and BMDMs isolated from both WT and NLRP6 KO mice following infection with *S*. *aureus* (MOI:10). Our results indicate that neutrophils, but not macrophages, from NLRP6 KO mice had improved killing ability compared to WT cells as depicted by the reduction in intracellular CFUs in KO neutrophils (Fig 4A and S2A Fig). However, the rate of phagocytosis was similar in neutrophils from both groups (WT and KO) (S2B Fig). It is known that neutrophils use NADPH oxidase-dependent reactive oxygen species (ROS) to kill *S*. *aureus* intracellularly [17–19]. To determine if differences in killing ability of WT and NLRP6 KO neutrophils is due to an alteration in ROS production, we compared ROS production by neutrophils from WT and KO mice after infection with *S*. *aureus* and found that KO neutrophils produced more ROS than WT neutrophils (Fig 4B). To confirm these *in vitro* findings, we assessed the expression of NADPH oxidase components in lung homogenates from infected mice by western blotting. The expression of p47^phox^, p67^phox^, and gp91^phox^ was increased in the lungs from NLRP6 KO mice compared to those from WT mice, supporting the finding of higher ROS production by KO neutrophils (Fig 4C).

IFN-γ has been shown to activate phagocytic cells during intra-pulmonary *S*. *aureus* infection [20]. Therefore, we measured the levels of IFN-γ secreted in the BALF of WT and NLRP6 KO mice after infection with *S*. *aureus*. Interestingly, we found that IFN-γ production was higher in KO mice compared to that of WT mice (Fig 4D). The production of IFN-γ requires activation of MAPK pathways [21]. Accordingly, we found higher MAPK activity in NLRP6 KO lungs than in WT lungs (Fig 4E). IFN-γ has been shown to induce ROS production in human mast cells during *Staphylococcal* infection [22]. Based on this observation, we assessed if IFN-γ contributes to the enhanced bacterial killing by neutrophils through induction of ROS. To this end, BMDNs from WT and KO mice were pre-treated with IFN-γ and subsequently infected with *S*. *aureus* (MOI of 10) followed by assessment of ROS production. Treatment of neutrophils with IFN-γ increased NADPH oxidase activity and ROS production (Fig 4F and 4G). The activation of phagocytes by IFN-γ involves activation of signal transducer and activator of transcription (STAT) proteins [23]. Supporting this observation, higher expression of phospho-STAT3 was found in the lungs of KO mice compared to WT (Fig 4E). Collectively, these results suggest that genetic ablation of NLRP6 increases bacterial killing by neutrophils through increased IFN-γ and ROS production, which are associated with higher NADPH oxidase activity.

### IFN-γ mediates bacterial clearance in NLRP6 KO mice

**Fig 5:**
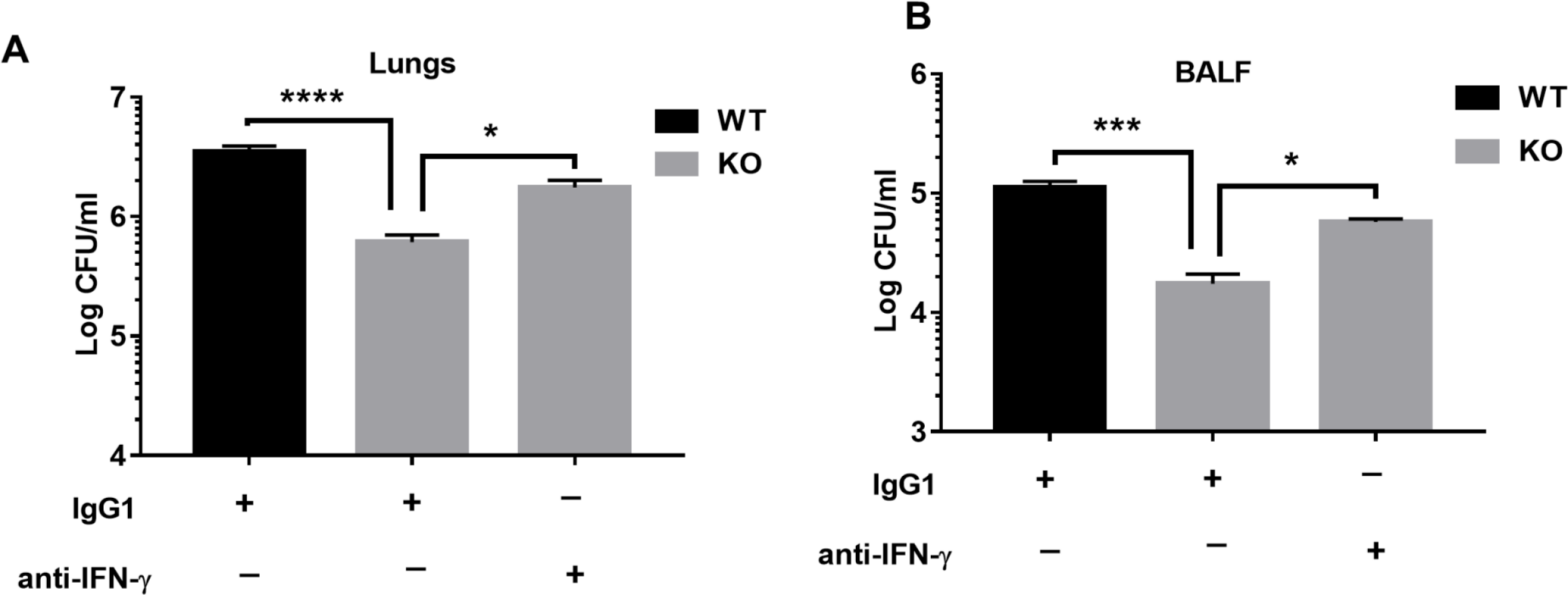
IFN-γ mediates bacterial clearance in NLRP6 KO mice during *S*. *aureus*-induced pneumonia. KO mice (n = 6-8/group) were treated with either anti-IFN-γ antibody (100 μg/mouse, i.p.) or isotype control antibody 12 hours prior to infection with *S*. *aureus* (5 X 10^7^ CFU/mouse). WT mice were treated with equal volume of isotype control antibody 12 hours prior to infection with *S*. *aureus* (5 X 10^7^ CFU/mouse). Twelve-hours post-infection, mice were euthanized to collect lungs and BALF. Bacterial burden in the **(A)** lungs, and **(B)** BALF were compared. All figures are representative of three independent experiments. *, p<0.05, **, p<0.01, *** p<0.001.

Our *in vivo* experiments demonstrate that NLRP6 KO mice exhibit improved bacterial clearance (Fig 2B–2D), higher neutrophil accumulation, as well as enhanced IFN-γ production (Figs 3C and 4D). Moreover, our *in vitro* experiments using BMDNs demonstrate that IFN-γ enhance bacterial killing by neutrophils through increased ROS production (Fig 4G). Therefore, we examined if blocking IFN-γ hinders bacterial clearance in the KO mice following infection with *S*. *aureus*. In this context, we administered anti-mouse IFN-γ antibody (100 μg/mouse i.p.) to one group of the KO mice and a similar volume of isotype antibody to another group of mice 12 hours before infection with *S*. *aureus*. Blocking of IFN-γ in the KO mice increased the bacterial burden in the lungs and BALF suggesting that IFN-γ mediates bacterial clearance in the KO mice (Fig 5A and 5B).

### Natural killer and CD4^+^T cells are the primary sources of IFN-γ

**Fig 6:**
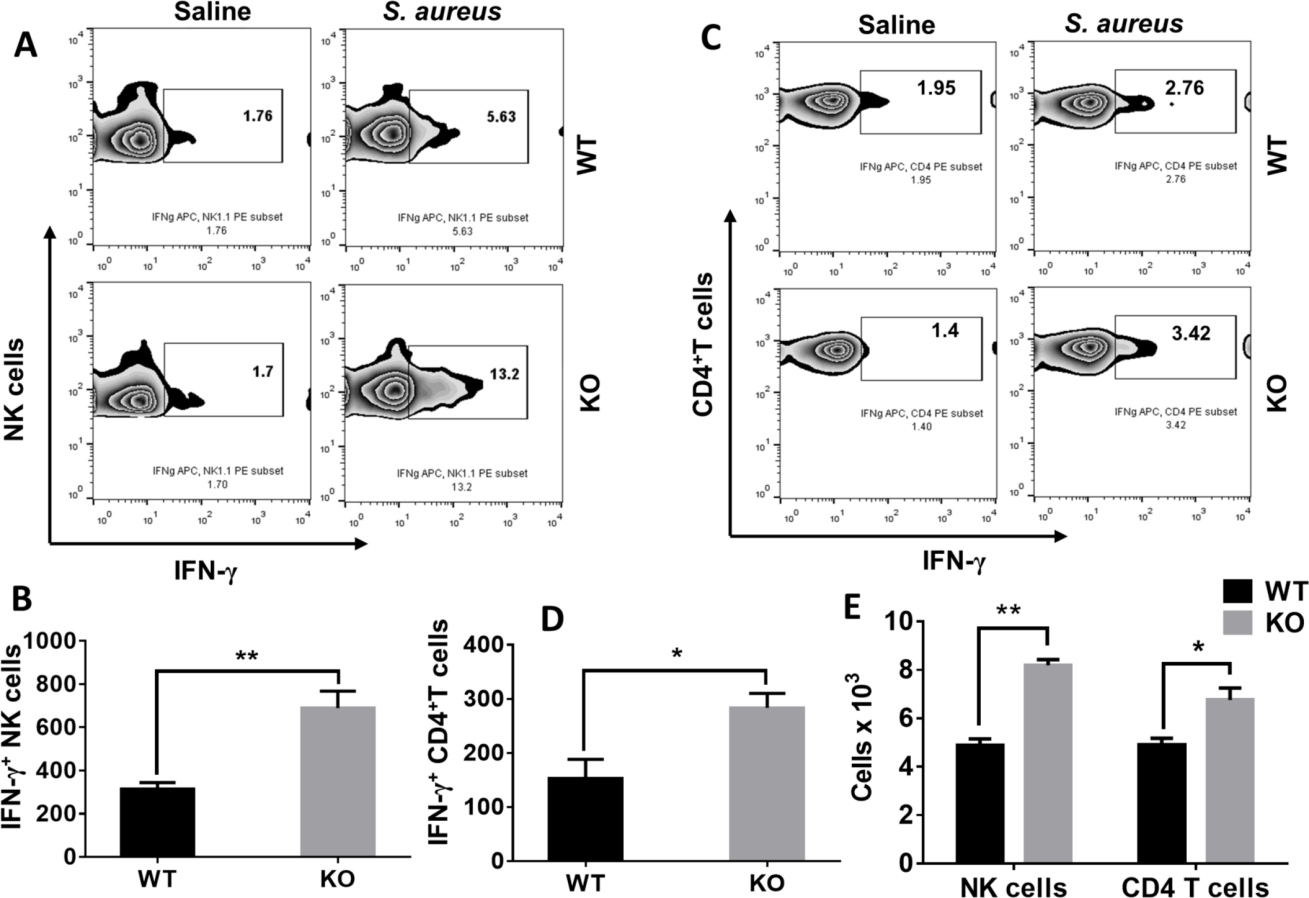
NK cells and CD4^+^ T cells are the major sources of IFN-γ production during pulmonary *S*. *aureus* infection. WT and KO mice (N = 6-8/group) were infected with *S*. *aureus* (5 X 10^7^ CFU/mouse). Twelve-hours post-infection, mice were euthanized to harvest lungs. Single cell suspensions obtained through digestion of lungs were treated with PMA/Ionomycin and GolgiStop for 5 hours and then stained for flow cytometry. **(A)** Representative zebra plot showing CD3^-^ IFN-γ ^+^NK 1.1^+^ cells. **(B)** Quantification of **A**. **(C)** Representative zebra plot showing CD3^+^ IFN-γ^+^ CD4^+^T cells. **(D)** Quantification of **C**. **(E)** WT and KO mice (N = 6-8/group) were infected with *S*. *aureus* (5 X 10^7^ CFU/mouse). Twenty-four hours post-infection, mice were euthanized to collect lungs tissues. Single cell suspensions obtained after lung digestion were stained to estimate NK and CD4 T cells using flowcytometry. The figures shown above are representatives of three independent experiments. *, p<0.05, **, p<0.01.

We next sought to identify the cellular sources of IFN-γ during *S*. *aureus* infection. To this end, we performed flow cytometric analysis of lung cells from WT and KO mice following infection with *S*. *aureus* and found that NLRP6 KO mice had more IFN-γ-positive NK and CD4^+^T cells (Fig 6A–6D). In this regard, Nguyen *et al* reported NK cells as the major source of IFN-γ during *S*. *aureus* infection [20]. We also found CD8^+^T cells and γδT cells produce IFN-γ, although there was no difference between WT and KO mice in the total number of IFN-γ-positive CD8 or γδT cells in the lungs (S3A–S3D Fig). To determine whether increased IFN-γ production in the KO mice is due to higher MAPK activity in NK and CD4 T cells, we isolated these cells from the lungs of WT and KO mice and treated them with MAPK inhibitor prior to infection with *S*. *aureus*. However, blocking MAPK did not reduce IFN-γ secretion by NK and CD4 T cells (S3E Fig and S3F Fig). Furthermore, we measured the number of NK and CD4 T cells in the lungs after infection through flow cytometry and found that the KO mice had more NK and CD4 T cells accumulated in the lungs compared to that of WT mice (Fig 6E). These results together suggest that increased IFN-γ observed in the KO mice is due to enhanced numbers of NK and CD4 T cells recruited during infection.

### Deletion of NLRP6 decreases *S*. *aureus*-induced pyroptosis and necroptosis

**Fig 7:**
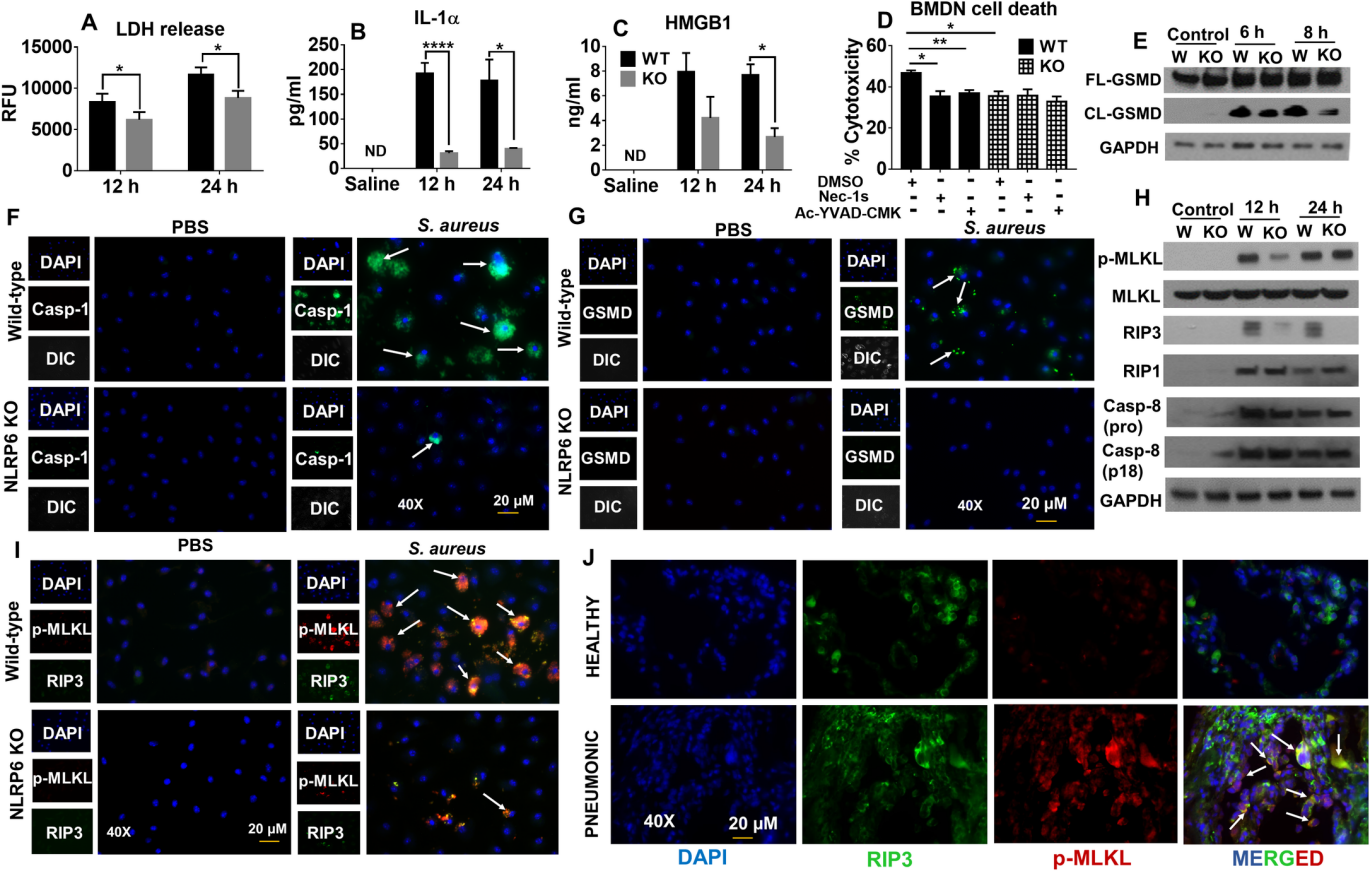
NLRP6^-/-^ mice display reduced pyroptosis and necroptosis during *S*. *aureus* induced pneumonia. WT and KO (N = 6-8/group/time point) mice were infected with a sublethal dose of *S*. *aureus* (5 X 10^7^ CFU/mouse). **(A)** LDH released in BALF, **(B)** IL-1α in BALF, and **(C)** HMGB1 in serum were measured. **(D)** BMDNs from WT and KO mice were isolated and pre-treated with either Ac-YVAD-CMK (100μg/ml), Nec-1s (300 μM), or an equivalent amount of DMSO for 30 minutes prior to infection with *S*. *aureus* (MOI 50). Cytotoxicity was measured 2 hours post-infection. **(E)** Expression of cleaved gasdermin-D in BMDMs obtained from WT and KO mice after infection with *S*. *aureus* for 8 hours. **(F)** BMDMs from WT and NLRP6 KO mice were infected with *S*. *aureus* (MOI 50) for 8 hours. Caspase-1 activation was observed through fluorescence microscopy. The upper panel shows WT and the lower panel shows KO macrophages. Caspase-1 positive cells are shown by white arrowheads. **(G)** BMDMs from WT and NLRP6 KO mice were infected with *S*. *aureus* as in **F**. Gasdermin-D positive cells were observed through fluorescence microscopy. The upper panel indicates WT and lower panel indicates KO macrophages after infection with *S*. *aureus*. **(H)** Western blots to show expression of phospho-MLKL, RIP3, RIP1, and cleaved caspase-8 in lungs homogenates obtained from *S*. *aureus*-infected mice. WT and KO mice (N = 6-8/group/time-point) were infected with a sublethal dose of *S*. *aureus* (5 X 10^7^ CFU/mouse, i.t). 12 and 24 h post-infection, mice were euthanized to collect lungs and processed for western blotting. **(I)** Fluorescent microscopy showing expression of molecules involved in necroptosis in macrophages after infection with *S*. *aureus*. BMDMs obtained from WT and KO mice were infected with *S*. *aureus* (MOI 50) for 8 hours and then stained for phospho-MLKL (red) and RIP3 (green) antibody. Necroptotic cells are represented as orange (indicated by white arrowheads). **(J)** Human pneumonic lungs showing necroptosis. Lungs tissue sections from healthy and pneumonic patients were deparaffinized and stained with phospho-MLKL, RIP3, and DAPI. Tissue undergoing necroptosis is shown by white arrowheads. Immunofluorescence pictures were taken at 40X. All figures are representative of three independent experiments. Ac-YVAD-CMK: caspase-1 inhibitor, Nec-1s: Necrostatin-1s, CL-GSMD: Cleaved Gasdermin-D, FL-GSMD: Full-length Gasdermin-D, WT: *, p<0.05, **, p<0.01, and *** p<0.001.

Since we found decreased neutrophil recruitment and increased protein leakage in the lungs of WT mice compared to KO mice (Figs 3C and 2E), we hypothesized that cell death in the lungs may be responsible for these results during *S*. *aureus* infection. In this regard, LDH, IL-1α, and HMGB-1 are intracellular molecules released exclusively after cell death and are thus termed *alarmins* [24, 25]. Therefore, we measured the levels of these alarmins in an *in vivo* setting and found their expression to be increased in WT mice compared to NLRP6 KO mice. This suggests that NLRP6 enhances inflammatory modes of cell death during *S*. *aureus*-induced pneumonia (Fig 7A–7C). We also measured the extent of cell death in BMDNs following infection with *S*. *aureus*. Neutrophils from WT mice exhibited increased cell death, as seen by increased cytotoxicity (LDH release), compared to that of KO neutrophils (Fig 7D). Next, to determine the nature of cell death, BMDNs from WT and KO mice were pre-treated with either Ac-YVAD-CMK (Caspase-1 inhibitor) or Necrostatin-1s (Nec-1s, necroptosis inhibitor) and infected with *S*. *aureus*. Pre-treatment of neutrophils with Ac-YVAD-CMK or Nec-1s reduced the cell death in WT neutrophils suggesting that the nature of cell death is both pyroptosis and necroptosis (Fig 7D). In this regard, it is reported that caspase-1 and gasdermin-D mediate pyroptosis during bacterial infection [26–28]. To validate that NLRP6 induces pyroptosis, we assessed the expression of caspase-1 and gasdermin-D in BMDMs from WT and NLRP6 KO mice after infection with *S*. *aureus* (MOI of 20) for 8 hours. Both cleaved caspase-1 (Fig 1F) and gasdermin-D (Fig 7E) expression were higher in WT mice compared to KO mice. Also, we performed immunofluorescence microscopy on BMDMs to quantify caspase-1 and gasdermin-D expression and found increased caspase-1 and gasdermin-D- positive cells in BMDMs from WT mice compared to NLRP6 KO mice (Fig 7F and 7G; S4A and S4B Fig).

It has been reported that *S*. *aureus* induces pathology in the lungs by a distinct cell death mechanism known as necroptosis [29]. Receptor-interacting-serine-threonine kinase-1 (RIP1), RIP3, and mixed lineage kinase-domain like protein (MLKL) are the core protein kinases that initiate and execute necroptosis [29–31]. Recently, caspase-8 has been shown to negatively regulate necroptosis during *Salmonella* infection in an enteritis model [32]. Thus, to explore whether NLRP6 can enhance necroptosis in the lungs during *S*. *aureus* infection, we assessed the expression of phospho-MLKL RIP1, RIP-3, and caspase-8 in lung homogenates obtained from *S*. *aureus*-infected WT and NLRP6 KO mice through western blotting. Interestingly, both phospho-MLKL and RIP-3 were higher in the lungs of WT mice compared to NLRP6 KO mice (Fig 7H). To further confirm this finding at the cellular level, we infected both WT and NLRP6 KO BMDMs with *S*. *aureus* for 8 hours and quantified necroptosis using immunofluorescence microscopy. The immunofluorescence assay also revealed more phospho-MLKL and RIP-3-positive cells in WT macrophages compared to NLRP6 KO macrophages after *S*. *aureus* infection, confirming that NLRP6 increases necroptosis (Fig 7I and S4C Fig).

Since *S*. *aureus* has been shown to induce necroptosis in human cells, such as THP-1 cell lines [29], we examined if activation of the necroptosis pathway occurs in lungs of human patients during pneumonia. Immunofluorescence microscopy conducted on lung sections obtained from pneumonia patients displayed more necroptosis, as evidenced by increased phospho-MLKL and RIP-3 expression, compared to that of healthy control lungs (Fig 7J).

### Blocking pyroptosis and necroptosis in WT mice augments host defense

**Fig 8:**
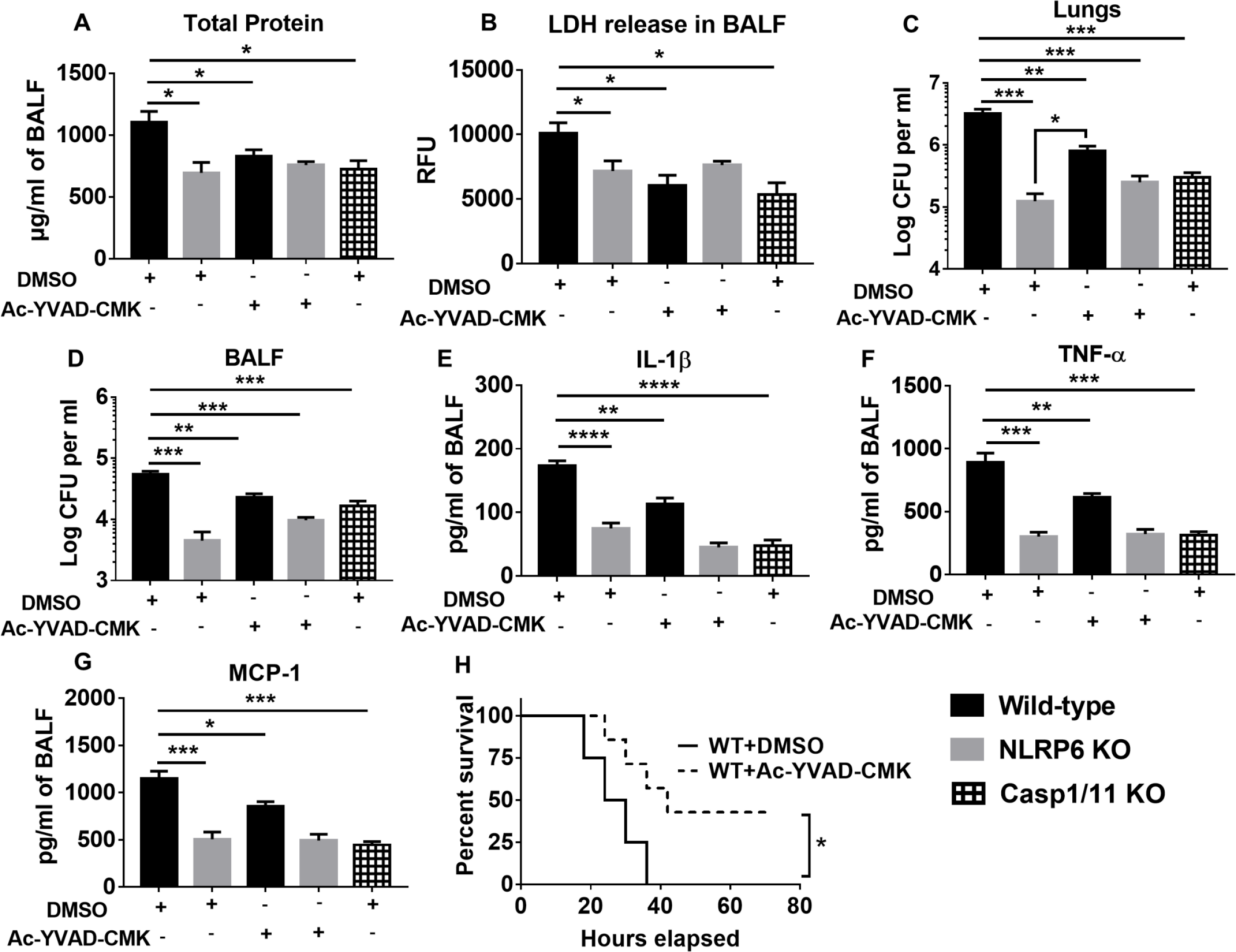
NLRP6-mediated pyroptosis is detrimental during pulmonary *S*. *aureus* infection. WT and KO mice (N = 6-8/group) were administered either Ac-YVAD-CMK (100 μg/mouse) or an equal amount of DMSO 12 hours prior to infection with a sublethal dose of *S*. *aureus*. Caspase-1/11 double knock out mice (N = 6-8/group) received an equal amount of DMSO 12 hours prior to infection with a sublethal dose of *S*. *aureus*. Mice were euthanized 24 hours post-infection to collect BALF and lungs. **(A)** Total protein and **(B)** LDH release in the lungs, bacterial burden in **(C)** lungs and **(D)** BALF were measured. **(E)** IL-1β, **(F)** TNF-α, and **(G)** MCP-1 in the BALF were measured through standard ELISA procedure. **(H)** WT mice (N = 15/group) were treated with either Ac-YVAD-CMK (100 μg/mouse 12 hours prior to infection with *S*. *aureus*), or an equivalent amount of DMSO prior to infection with a lethal dose of *S*. *aureus* (2 X 10^8^ CFU/mouse, i.t.). Survival was monitored up to 80 hours post infection. Each figure is a representative of at least three independent experiments. Ac-YVAD-CMK: caspase-1 inhibitor, TNF-α: Tumor necrosis factor-α, MCP-1: Monocyte chemoattractant protein-1, *, p<0.05, **, p<0.01, *** p<0.001, and **** p<0.0001.

We found that NLRP6 enhances both pyroptosis and necroptosis pathways during pulmonary *S*. *aureus* infection (Fig 7A–7I). Because pyroptosis has been found to be beneficial for bacterial clearance during intracellular bacterial infections [28], we examined whether pyroptosis is advantageous during *S*. *aureus* infection. Blockade of pyroptosis in mice by intra-peritoneal administration of Ac-YVAD-CMK (caspase-1 inhibitor) 12 hours prior to infection with *S*. *aureus* resulted in reduced pulmonary leakage, LDH release, and bacterial burden in lungs and BALF (Fig 8A–8D). In addition, blockade of pyroptosis suppressed cytokine secretion and enhanced survival of WT mice (Fig 8E–8H), confirming that pyroptosis is detrimental during *S*. *aureus* infection. It should be noted that the bacterial burden in WT mice receiving Ac-YVAD-CMK was still high when compared with NLRP6 KO mice (Fig 8C), suggesting that an NLRP6-dependent, but caspase-1-independent, mechanism also exists to increase susceptibility to *S*. *aureus* infection.

**Fig 9:**
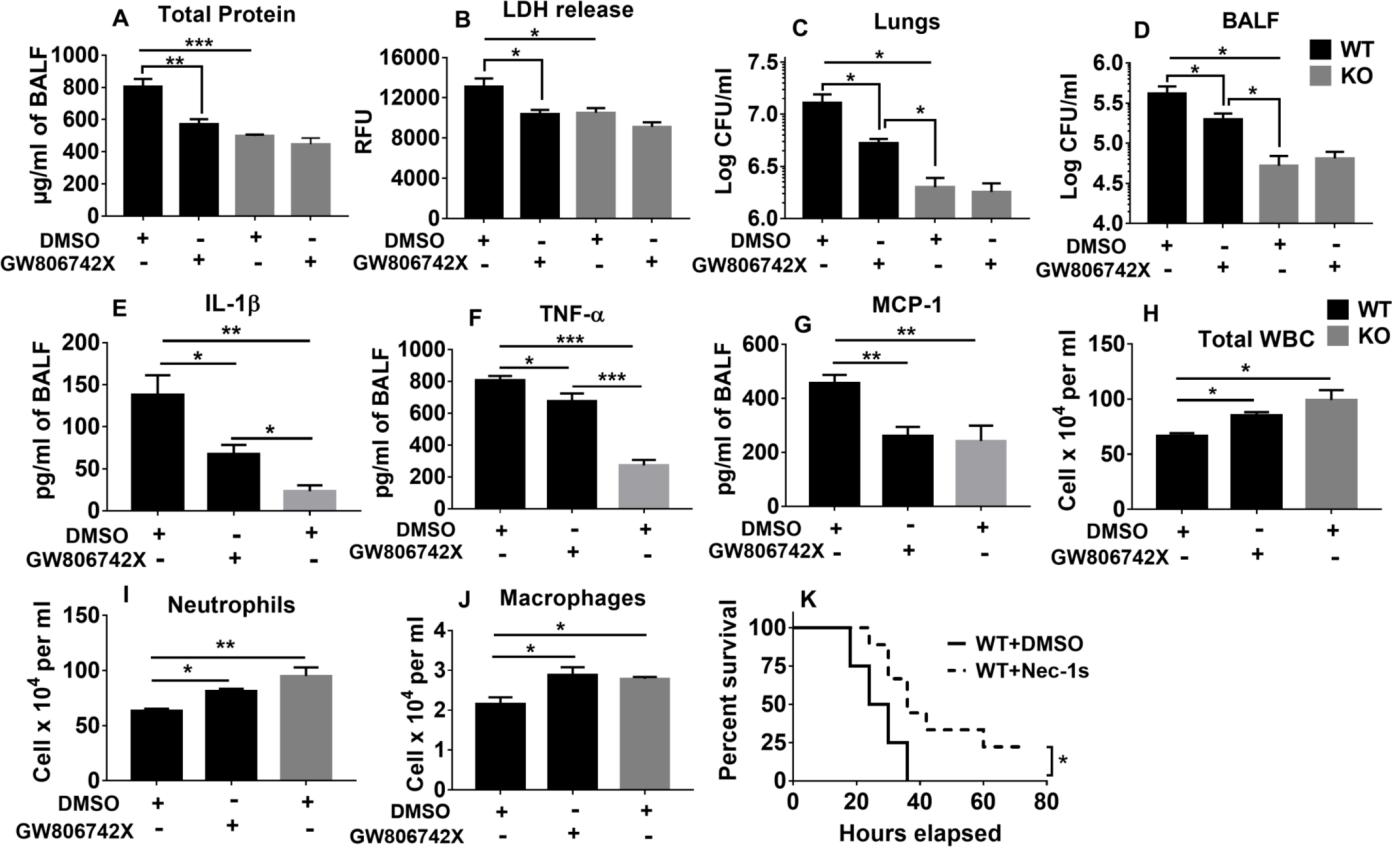
Blocking NLRP6-mediated necroptosis in WT mice improves host defense during *S*. *aureus* infection. WT and KO mice (N = 6-8/group) were treated with either GW806742X (100 μl of 100 μM solution, i.p., 12 hours prior to infection) or an equivalent amount of DMSO prior to infection with a sublethal dose of *S*. *aureus*. Mice were euthanized 24 hours post infection to collect lungs and BALF. **(A)** Total protein and **(B)** LDH release in the BALF, bacterial burden in **(C)** lungs and **(D)** BALF were measured. **(E)** IL-1β, **(F)** TNF-α, and **(G)** MCP-1 in the cell-free BALF were measured. **(H)** Total leukocytes, **(I)** neutrophils, and **(J)** macrophages in the BALF was determined. **(K)** WT mice (N = 15/group) were treated with either Nec-1s (300 μg/mouse) or an equivalent amount of DMSO, 18 hours before and at the time of infection with *S*. *aureus* (2X10^8^ CFU/mouse). Survival was monitored for up to 80 hours. Each figure is representative of three independent experiments. GW806742X: MLKL inhibitor, Nec-1s: Necrostatin-1s. *, p<0.05, **, p<0.01, and *** p<0.001.

Next, to confirm that NLRP6-mediated necroptosis is detrimental for host defense, we blocked necroptosis using an MLKL inhibitor (GW806742X) 12 hours before infection with *S*. *aureus*. There was a decrease in total protein and LDH release in the BALF of WT mice treated with GW806742X, which was comparable to that seen in NRLP6 KO mice (Fig 9A and 9B). Moreover, blocking necroptosis reduced the bacterial burden in lungs and BALF and ameliorated the inflammatory cytokine levels in WT mice (Fig 9C–9G). Also, leukocyte recruitment (neutrophils and macrophages) and survival were also increased in WT mice treated with necroptosis inhibitor (Fig 9H–9K). Inhibition of necroptosis using a RIP-1 inhibitor (Nec-1s) also reduced total protein leakage and LDH release in the BALF (S5 Fig). Collectively, these results reveal that NLRP6-mediated pyroptosis and necroptosis are detrimental to bacterial clearance and host survival during pulmonary *S*. *aureus* infection.

## Discussion

Lung diseases induced by Gram-positive pathogens are an important cause of morbidity and mortality in both immunocompetent and immunocompromised individuals [13, 33]. Although antibiotics decrease the mortality rates of bacterial pneumonia, the efficacy is somewhat limited due to the substantial number of immunocompromised individuals, growing number of elderly patients, and the rise of multi-antibiotic resistant bacterial strains. Thus, alternative therapeutic approaches, including the manipulation of host signaling events, are needed. However, detailed understanding of the host innate immune response is critical for the design of potential therapeutic interventions. Because the lung is continuously exposed to pathogens and their virulence factors, this organ possesses a multifaceted host defense system. Moreover, a successful immune response in the lung is critical for efficient clearance of microbial pathogens and therefore, the innate immune system possess germline-encoded pattern-recognition receptors.

The NOD-like receptors (NLRs), including inflammasomes, are specialized cytosolic pattern-recognition receptors/sensors necessary for clearance of invading cytosolic pathogens. Under normal homeostatic conditions, the NLRP6 inflammasome is highly expressed in intestinal epithelial cells [9, 14, 34, 35] where it co-localizes with ASC and caspase-1 [9]. It is also expressed in immune cells including neutrophils, T-cells, macrophages, and dendritic cells [7]. Despite high expression of NLRP6 in the lower respiratory tract, the role of NLRP6 in lung inflammation has not previously been explored. In the current study, we demonstrate that NLRP6 is upregulated in neutrophils, macrophages, and epithelial cells in the lungs of human pneumonia patients. Furthermore, NLRP6 is upregulated in myeloid and non-myeloid cells in the lungs and co-localizes with ASC in a mouse model of pulmonary *S*. *aureus* infection. We also found that the important virulence factor, α-hemolysin, can activate the NLRP6 inflammasome.

The immune response to *S*. *aureus* is manifested by vascular leakage, neutrophil recruitment into the alveolar space, and upregulation of cytokines and chemokines [5, 6]. The current study demonstrates that the NLRP6 inflammasome increases susceptibility to *S*. *aureus*-induced lung infection. Delving into the mechanisms underlying this, we found that NLRP6 dampens NK cell-mediated IFN-γ secretion thereby hindering ROS-dependent bacterial clearance by neutrophils. Moreover, our study identifies NK cells and CD4-T cells as the primary source of IFN-γ during acute pulmonary *S*. *aureus* infection. In agreement with these findings, studies of *Listeria* and *Salmonella* infections [7] have also shown that NLRP6 signaling is detrimental to host defense. Nonetheless, in a non-infectious model, the NLRP6 inflammasome was found to be important for epithelial self-renewal, proliferation, and mucus secretion, which were essential for protection against chemical-induced colitis [9, 34].

Studies from different groups have shown that NLRP6 inflammasome regulates gut microbiota composition [7, 9, 14]. NLRP6 KO mice were found to have different microbiota configuration which make them more susceptible to chemical-induced colitis compared to the WT mice [14]. In contrast, recent studies have demonstrated that NLRP6 and ASC-related inflammasome do not regulate gut microbiota composition [36, 37]. Although it remains debatable whether the NLRP6 inflammasome influence gut microbiome, the reported difference in microflora composition in the KO mice can be nullified by co-housing the mice together with WT for 4 weeks [7]. In addition to colitis, microbiota have been shown to influence various disease conditions such as rheumatoid arthritis [38], diabetes [39], inflammatory bowel disease [40], and colorectal cancer [41]. In our study, however, co-housing of WT with NLRP6 KO mice did not change the resistant phenotype of the KO mice against *S*. *aureus*. In this context, similar report has been demonstrated by Anand *et al*, showing that co-housing does not alter NLRP6 mediated immune mechanism during *Salmonella* and *Listeria* infection [7].

It is widely accepted that hematopoietic and non-hematopoietic (stromal) cells in the lung produce numerous proinflammatory mediators, including cytokines and chemokines. Although hematopoietic cells secrete chemokines or neutrophil chemo-attractants, including CXCL1/KC and CXCL2/MIP-2, the stromal cells (alveolar epithelial cells) secrete other neutrophil chemo-attractants, such as CXCL5/LIX and CXCL15/lungkine [42]. Our observations indicate that NLRP6 from both cell types contributes to the enhanced susceptibility to *S*. *aureus*-induced pneumonia. These conclusions are consistent with previous studies of the role of hematopoietic and non-hematopoietic cells in the context of bacterial infections in the lungs. In this context, NLRP6 in both hematopoietic and non-hematopoietic cells increases susceptibility to *Listeria* and *Salmonella* infections [7]. Similarly, CXCL1/KC secreted by both hematopoietic and stromal cells was found to be crucial for bacterial elimination and neutrophil accumulation in the lungs following *Klebsiella pneumoniae* infection [43]. Nevertheless, it is clear from this investigation that neutrophil accumulation and function are critical for host protection against *S*. *aureus*.

Pyroptosis and necroptosis are two distinct inflammatory modes of cell death. While pyroptosis is mediated by caspase-1 [26–28] and executed by gasdermin-D [26, 27], necroptosis is regulated by RIP1, RIP3, and MLKL (caspase-1 independent) [29, 30]. Pyroptosis has been shown to play an essential role in limiting several intracellular bacterial infections such *Salmonella typhimurium*, *Legionella pneumophila*, and *Burkholderia thailandensis [28]*. However, extensive caspase-1 activation and subsequent pyroptosis have also been associated with the severity of several diseases such as myocardial infarction [44], inflammatory bowel disease [45], and endotoxic shock [46]. Pertaining to these observations, we report that during *S*. *aureus* infection, NLRP6-mediated pyroptosis is detrimental for host survival. Furthermore, blocking pyroptosis reduced the hyper-inflammatory milieu and subsequently increased survival suggesting that pyroptosis triggers exaggerated inflammation during *S*. *aureus* infection. This difference in the role of pyroptosis can be attributed to differences in pathogenic properties and life styles of bacterial pathogens. While *S*. *aureus* is predominantly an extracellular pathogen, studies have shown that it can also survive intracellularly [47] and can resist anaerobic conditions [48]. Necroptosis induced by *S*. *aureus* is responsible for pathology in the lung [29]; however, its relationship with inflammasomes was previously unknown. Although ASC and NLRP3 have been linked with pore-forming toxin-induced necroptosis [31], the precise role of inflammasomes in the induction of necroptosis is not clear. In this study, we used both *in vivo* and *in vitro* experiments to show that NLRP6 mediates necroptosis of immune cells during acute pulmonary *S*. *aureus* infection. Moreover, *S*. *aureus* exploits NLRP6 to drive necroptosis, which is accompanied by an intense inflammatory response and loss of macrophages and neutrophils. It is possible that reduced cell death in NLPR6 KO mice attributed to higher leukocyte accumulation in the lungs of these mice. Since TNF-α has been shown to induce necroptosis [49, 50], the reduction of TNF-α found in the NLRP6 KO mice suggests that NLRP6 can trigger necroptosis via the TNF-α pathway. However, more comprehensive future studies will be needed to identify the detailed molecular mechanisms underlying NLRP6-mediated necroptosis. Future studies are also needed to determine whether other toxins or virulence factors produced by *S*. *aureus* can also activate the NLRP6 inflammasome.

In conclusion, the present study reveals the detrimental role of NLRP6 during *S*. *aureus* pneumonia (S6 Fig). Furthermore, NLRP6 in both hematopoietic and resident lung compartments contributes to *S*. *aureus*-induced lung inflammation. Not only does NLRP6 subdue neutrophil function by dampening IFN-γ and ROS production, it also triggers pyroptosis and necroptosis in the lungs that may lead to hyper-inflammation, loss of neutrophils, and mortality. However, future studies are essential to determine whether NLRP6 interacts with other inflammasomes such as NLRP3 and/or NLRC4 to induce pyroptosis and necroptosis during *S*. *aureus* infection. Comprehensive studies using specific double- or triple-KO mice would be useful to delineate these interactions in a conclusive manner. Furthermore, extending upon our findings, we propose that functional single nucleotide polymorphisms in human NLRP6 may have effects on host defense mechanisms against gram-positive microbes.

## Materials and methods

### Ethics statement

Mouse experiments were conducted in accordance with the recommendations in the Guide for the Care and Use of Laboratory Animals of the National Institutes of Health. Animal protocols were approved by the Institutional Animal Care and Use Committee (IACUC) at Louisiana State University (protocol number 16–072). All animal experiments were performed in a manner to ensure minimal pain and distress. *Nlrp6*^*-/-*^, *Asc*^*-/-*^, and *Caspase-1/11*^*-/-*^ were obtained from the Millennium Pharmaceuticals (Cambridge, MA) whereas C57Bl/6 mice were obtained from Taconic (Rensselaer, NY) and Jackson (Bar Harbor, ME) Laboratories. THP-1 (human monocytic) cells and HL-60 (human neutrophil-like) cells were purchased from ATCC (Manassas, VA). Lysates of human healthy control tissue and pneumonic lung tissue were obtained from Novus Biologicals, CO.

### Immunofluorescence microscopy

Immunofluorescence microscopy of lung sections was performed as described previously [51]. Human lung sections from lungs without evidence of infection or injury (control) or from patients who died due to ALI/ARDs (pneumonic) were used from BioChain Institute Inc. (Newark, CA). Mouse lung sections were from saline-challenged or *S*. *aureus*-infected mice. Lung sections were incubated with anti-NLRP6 (Abgent, CA) and antibodies for surface markers including anti-lipocalin Ab for neutrophils (R&D Systems, MN), or anti-CD68 Ab for macrophages (BioLegend, CA), and anti-CD326 Ab for alveolar epithelial cells (BioLegend, CA). For mouse lung sections, we used antibodies for surface markers including anti-Ly6G for PMNs (BioLegend, CA), anti-F4/80 for macrophages (BioLegend, CA), and anti-CD326 for alveolar epithelial cells along with anti-NLRP6 Ab (Sigma, MO). Appropriate Alexa-conjugated secondary antibodies (Invitrogen, CA) were used. For detection of necroptosis and pyroptosis through an immunofluorescence assay, antibodies against mouse NLRP6 and ASC (Sigma, MO), p-MLKL, (Abcam, MA), RIP3 (Cell signaling, MA), caspase-1 (Adipogen, CA), and gasdermin-D (Santa cruz, CA) were used. Excess antibodies were washed off, and the cells were labeled with secondary antibodies, such as mouse IgG/IgM (H+L) Alexa fluor 488, 568 (Invitrogen, CA). Images were obtained using an Axiocam digital camera (Zeiss, NY) connected to a Zeiss Axioskop 2 Plus microscope.

### Immunoblotting and cytokine measurement

BMDMs or lungs were harvested at designated time points and homogenized in PBS containing 0.1% Triton X-100 (phosphatase and protease inhibitor cocktail added). After centrifugation the supernatants were used for immunoblotting. Total protein content in the supernatant was measured using a BCA protein assay kit (Thermofisher, NY) to ensure that equal amounts of proteins were loaded onto 10% SDS-PAGE gels. Proteins were transferred to polyvinylidene fluoride membrane according to the protocol provided by Bio-Rad. Appropriate primary antibodies against mouse NLRP6 (Sigma, MO), phospho-MLKL (Abcam, MA), RIP3, RIP1, P47^phox,^ P67^phox,^ gp91^phox^, phospho-p38 MAPK, phospho-JNK, phospho-Stat3, caspase-8, GAPDH (Cell Signaling, MA), caspase-1 (Adipogen), and gasdermin-D (Santa Cruz, CA) were added to the membrane and incubated overnight at 4^0^ C. Appropriate secondary antibodies were used, and the films were developed using ECL plus western blot detection system (ThermoFisher, NY). IL-1β, TNF-α, IFN-γ, IL-1α, and IL-6 were measured in BALF supernatants by ELISA according to the manufacturer’s protocol (eBioscience, CA).

### Animals

Eight to twelve-week-old WT mice (C57BL/6 background) were used. Equal age- and gender-matched NLRP6 KO, ASC KO, and Caspase-1/11 DKO mice on the C57BL/6 background were used throughout the experiments. Mice were kept on a 12:12 hour light/dark cycle under specific pathogen-free condition with free access to food and water. All animal experiments were approved by the Institutional Animal Care and Use Committee (IACUC) at Louisiana State University.

### Pneumonia model

To induce pneumonia, mice were anesthetized using ketamine (100 mg/kg) and xylazine (5 mg/kg) prior to intratracheal inoculation of *S*. *aureus* (USA 300 strain). A small midline incision was made on the ventral aspect of the neck and excess fat was separated to expose the trachea. Fifty microliters of bacterial suspension containing 5X10^7^ CFU of log phase *S*. *aureus* in isotonic saline was injected into the lungs by piercing trachea using a 28.5-gauge needle. At 12- and 24-hours post-infection, mice were euthanized to collect BALF, lungs, and liver for quantification of bacterial burden and leukocyte recruitment. BALF and homogenized organs were serially diluted and plated onto Tryptic soy agar (TSA) plates for bacterial enumeration. For survival experiments, we used 2 X 10^8^ CFUs/mouse of *S*. *aureus* and observed survival for 12 days post-infection.

### BALF collection

BALF was collected as described in our previous publication [51]. Briefly, after specific time points, mice were humanly euthanized, and the trachea was exposed. Using a 20-gauge catheter, 0.8 ml of PBS (heparin and dextrose added) was instilled inside the lungs and collected in a clean tube. The process was repeated a total of four times so that a minimum of 2.8–3 ml of BALF was collected from each mouse. Total leukocyte count was performed in a hemocytometer using 10 μl of BALF and the differential count was done under light microscopy using cytospin slides stained with DiffQuik reagent. The remaining cell-free BALF was preserved at -80^o^ C for cytokine analysis.

### Co-housing experiments

Co-housing experiments were performed as described by Anand et al [7]. In brief, age and sex matched WT and NLRP6 KO mice were co-housed together in 1:1 ratio for 4 weeks. After co-housing, WT and the KO mice were infected with *S*. *aureus* (i.t.) and euthanized 24 hours post-infection to measure the bacterial burden.

### Bone marrow chimeras

Bone marrow chimeras were generated as described previously [51, 52]. In brief, the recipient mice were lethally irradiated with a 1000-rad inoculum from a cesium source. Bone marrow cells collected from healthy donor mice were injected into recipient mice via tail vein (8 million cells per mouse). The chimeric mice were kept under 0.2% neomycin sulfate treatment for 15 days after transplantation. After 8 weeks of transplantation, the chimeric mice were infected with 5X 10^7^ CFU of *S*. *aureus*. The mice were euthanized at 24-hour post-infection to estimate cellular recruitment and bacterial burden in the lungs.

### Neutrophil depletion

For neutrophil depletion, mice were treated with 500 μg of anti-Ly6G antibody (clone 1A8, BioLegend, CA) [53] intraperitoneally 24 and 2 hours prior to infection with lethal inoculum of *S*. *aureus* (2 X 10^8^ CFUs/mouse). For IFN-γ inhibition, mice were treated with 100μg of IFN-γ antibody (BioXCell, NH) 12 hours prior to infection with *S*. *aureus*. For caspase-1 inhibition, mice were treated with 100 μg of caspase-1 inhibitor (Ac-YVAD-CMK, Cayman chemical, MI) 12 hours prior to infection with *S*. *aureus*. For MLKL inhibition, 100 μl of 100 μM MLKL inhibitor (GW806742X, Adipogen, CA) [31] was injected into each mouse i.p. 12 hours prior to infection with *S*. *aureus*. For RIP1 inhibition, mice were treated with 300 μg of necroptosis inhibitor (Nec-1/necrostatin-1s, Calbiochem, MA) [29] 18 hours before and at the time of bacterial infection, as published elsewhere [29]. Mice were euthanized to collect BALF and organs 24 hours post infection.

### MPO assay

For MPO activity assay, lungs obtained from WT and KO mice after infection were homogenized. The supernatants obtained after centrifugation were mixed with 50 mM potassium phosphate buffer (with 0.5% hexadecyltrimethylammonium bromide) in 1:5 ratio and then centrifuged again. The supernatants were transferred to a 96-well plate. Absorbance was measured using a spectrophotometer at 460 nm after adding hydrogen peroxide/O-dianisidine hydrochloride buffer.

### Neutrophil killing assay

The intracellular killing assay was performed as described [54] with slight modification. Briefly, bone marrow neutrophils from NLRP6 KO and WT mice were isolated, infected with *S*. *aureus* (MOI: 10), and treated with gentamicin for 30 minutes at designated time points post-infection (30 min, 60 min, 90 min, and 120 min) to kill extracellular bacteria. Cells were washed several times with sterile PBS to remove excess gentamicin and were then lysed with 0.1% triton X to release intracellular bacteria. The lysate was serially diluted with PBS, plated onto the TSA, and incubated at 37^0^ C overnight for bacterial load estimation.

### ROS detection

Total neutrophils, isolated from bone marrow of WT and NLRP6 KO mice using a magnetic negative selection cell isolation kit (STEMCELL Technologies, Vancouver, Canada), were infected with *S*. *aureus* (MOI 10). Total intracellular ROS production was quantified using a Fluorometric kit (AA Bioquest, CA). The effect of IFN-γ on ROS production was determined by pretreating neutrophils with either 20 ng/ml of recombinant mouse IFN-γ (R&D Systems, MN) or an equal volume of PBS for 30 minutes before infection with *S*. *aureus*. The total ROS production was quantified after 30 minutes of infection using a spectrophotometer.

### Measurement of cell death

BMDNs were isolated from WT and NLRP6 KO mice and pretreated with Nec-1s (300 μM) or Ac-YVAD-CMK (100 μg/ml) or DMSO for 30 minutes before infection with *S*. *aureus* (MOI: 20). The percentage of cytotoxicity in BMDNs and LDH release into the alveolar space after *S*. *aureus* infection were measured using the Cytotox-ONE^TM^ homogenous membrane integrity assay kit (Promega, WI). HMGB-1 was measured using a commercially available kit from Chondrex Inc, WA.

### Statistics

Data are represented as mean ± SEM. The Mann-Whitney test was used to compare the bacterial burden between two groups. Student’s t-test was used whenever the data were parametric in nature. We used one-way ANOVA followed by Bonferroni’s multiple comparison test wherever more than two groups were involved. All statistical analyses were performed using GraphPad Prism 7 software. The survival curve was analyzed using Log-rank (Mantel Cox) test. All experiments were performed thrice. Significant differences are indicated by * (p<0.05), ** (p<0.01), *** (p<0.001), and **** (p<0.0001).

## Supporting information

S1 Fig Activation of NLRP6 inflammasome during MRSA infection. **(A)** Bone marrow-derived macrophages (BMDM) from WT and KO mice were infected with MRSA. Six- and 12-hours post-infection, IL-18 in the supernatant was quantified. **(B)** BMDMs from WT and KO mice were primed with LPS for 4 hours and stimulated with ATP or Nigericin for one hour. IL-1β in the supernatant was quantified. **(C)** WT and KO mice (N = 5-6/group) were infected with S. aureus. Twelve hours post-infection, mice were euthanized to collect broncho-alveolar lavage fluid (BALF). IL-18 was measured in cell free BALF supernatants. **(D)** BMDMs obtained from WT and KO mice were infected with *S*. *aureus* and processed for immunofluorescence assay. Percentage co-localization of NLRP6 with ASC or caspase-1 was calculated after counting 300 cells. The figure shown is a representative figure of 3 separate experiments.
(DOCX)

S2 Fig The role of NLRP6 in bacterial killing in bone marrow-derived macrophages (BMDM). **(A)** BMDM from WT and KO mice were isolated and infected with MRSA (MOI: 10). Killing capacity was compared at indicated time points as described in methods section. **(B)** Rate of phagocytosis by bone marrow-derived neutrophils (BMDN). BMDN from WT and KO mice were isolated and rate of phagocytosis was measured after one hour using pHrodo red *S*. *aureus* bio-particles. Each figure is a representative figure of 3 independent experiments.
(DOCX)

S3 Fig Cellular source of IFN-γ in pulmonary MRSA infection. WT and KO mice (N = 9-11/group) were infected intra-tracheally with MRSA (5X10^7^ CFU/mouse). After 24 hours of infection, mice were euthanized to collect lungs. Single cell suspensions obtained from lungs were stimulated with PMA/ionomycin along with Brefeldin A for 4 hours and then stained intracellularly for IFN-**γ**. **(A)** Gating strategy to obtain cell positive for both **γ**δT cells and IFN-**γ**. **(B)** IFN-**γ** positive CD8^+^T cells. **(C)** Quantification of **A**. **(D)** Quantification of **B**. **(E)** NK cells and CD4 T cells **(F)** were isolated from WT and KO mice and pre-treated them with MAPK inhibitor (10μM) prior to infection with *S*. *aureus*. Cells were then stained intra-cellularly to detect IFN-**γ** positive NK and CD4 T cells. Percentage of IFN-**γ** positive cells are shown. Each figure is a representative of 3 independent experiments.
(DOCX)

S4 Fig Role of NLRP6 in necroptosis during MRSA infection in bone marrow-derived macrophages (BMDM). BMDM from WT and KO mice were infected with MRSA (MOI:50) for 8 hours and stained with antibodies against caspase-1, gasdermin-D, RIP3, and phopho-MLKL. Percentage of cells positive for caspase-1 (A), gasdermin-D (B), and RIP3 and p-MLKL (C) were calculated and represented in the graph. The graph is the representative of 3 independent experiments. *, p<0.05, **, p<0.01, ***, p<0.001.
(DOCX)

S5 Fig Effect of blocking of necroptosis in WT and KO mice during MRSA infection. WT and KO mice (N = 6-8/group) were treated with either Nec-1s or vehicle control 18 hours before and at the time of infection with MRSA. Twenty-four hours post-infection, mice were euthanized to measure total protein leakage **(A)** and extent of cell death **(B)** in the BALF. Each figure is a representative figure of at least 3 independent experiments. Nec-1s: Necrostatin-1s *, p<0.05.
(DOCX)

S6 Fig A scheme demonstrating how NLRP6 signaling induces mortality following pulmonary MRSA infection. Upon infection in the lungs, MRSA upregulates and activates NLRP6 inflammasome **(1)**. The activated NLRP6 inflammasome dampens IFN-**γ** production by NK-cells **(2)**. Since IFN-**γ** is important for NADPH oxidase activity, reduction of IFN-**γ** secretion leads to reduced activity of NADPH oxidase **(3)** and ultimately reduced ROS production **(4)** ultimately compromising the function of neutrophils **(5)**. Reduced ROS production by neutrophils leads to reduced bacterial clearance **(6)**. Upon activation, NLRP6 increases the activity of caspase-1 and gasdermin-D **(7)** thereby triggering pyroptosis **(8)**. Similarly, NLRP6 triggers necroptosis by upregulating RIP-3 and p-MLKL in the lungs **(9&10)**. Both of these inflammatory cell death mechanisms **(11)** lead to exaggerated inflammation and loss of immune cells **(12)**. Ultimately, there will be less bacterial clearance **(5)** and increased mortality **(13)**.
(DOCX)
