# Supplementary material for: NLRP6 negatively regulates pulmonary host defense in Gram-positive bacterial infection through modulating neutrophil recruitment and function
Source: PLoS Pathog. 2018 Sep 24;14(9):e1007308. doi: 10.1371/journal.ppat.1007308 (PMC6171945; doi:10.1371/journal.ppat.1007308)
Supplement: S1 Fig — (A) Bone marrow-derived macrophages (BMDM) from WT and KO mice were infected with MRSA. Six- and 12-hours post-infection, IL-18 in the supernatant was quantified. (B) BMDMs from WT and KO mice were primed with LPS for 4 hours and stimulated with ATP or Nigericin for one hour. IL-1β in the supernatant was quantified. (C) WT and KO mice (N = 5-6/group) were infected with S. aureus. Twelve hours post-infection, mice were euthanized to collect broncho-alveolar lavage fluid (BALF). IL-18 was measured in cell free BALF supernatants. (D) BMDMs obtained from WT and KO mice were infected with S. aureus and processed for immunofluorescence assay. Percentage co-localization of NLRP6 with ASC or caspase-1 was calculated after counting 300 cells. The figure shown is a representative figure of 3 separate experiments. (DOCX) [file ppat.1007308.s001.docx]

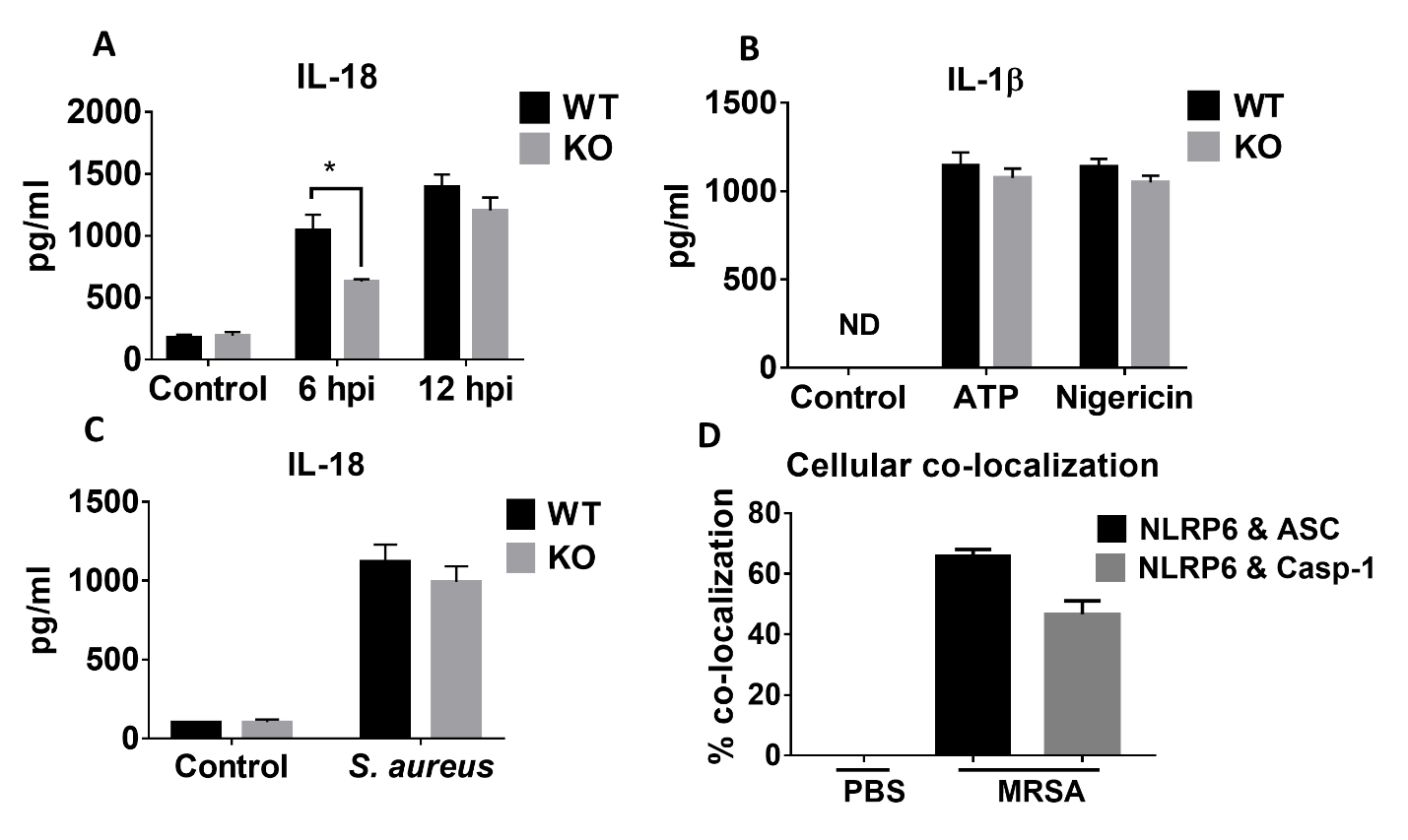
 **S1 Fig: Activation of NLRP6 inflammasome during MRSA infection. (A)** Bone marrow-derived macrophages (BMDM) from WT and KO mice were infected with MRSA. Six- and 12-hours post-infection, IL-18 in the supernatant was quantified. **(B)** BMDMs from WT and KO mice were primed with LPS for 4 hours and stimulated with ATP or Nigericin for one hour. IL-1β in the supernatant was quantified. **(C)** WT and KO mice (N=5-6/group) were infected with S. aureus. Twelve hours post-infection, mice were euthanized to collect broncho-alveolar lavage fluid (BALF). IL-18 was measured in cell free BALF supernatants. **(D)** BMDMs obtained from WT and KO mice were infected with *S. aureus* and processed for immunofluorescence assay. Percentage co-localization of NLRP6 with ASC or caspase-1 was calculated after counting 300 cells. The figure shown is a representative figure of 3 separate experiments.
