# Supplementary material for: NLRP6 negatively regulates pulmonary host defense in Gram-positive bacterial infection through modulating neutrophil recruitment and function
Source: PLoS Pathog. 2018 Sep 24;14(9):e1007308. doi: 10.1371/journal.ppat.1007308 (PMC6171945; doi:10.1371/journal.ppat.1007308)
Supplement: S2 Fig — (A) BMDM from WT and KO mice were isolated and infected with MRSA (MOI: 10). Killing capacity was compared at indicated time points as described in methods section. (B) Rate of phagocytosis by bone marrow-derived neutrophils (BMDN). BMDN from WT and KO mice were isolated and rate of phagocytosis was measured after one hour using pHrodo red S. aureus bio-particles. Each figure is a representative figure of 3 independent experiments. (DOCX) [file ppat.1007308.s002.docx]

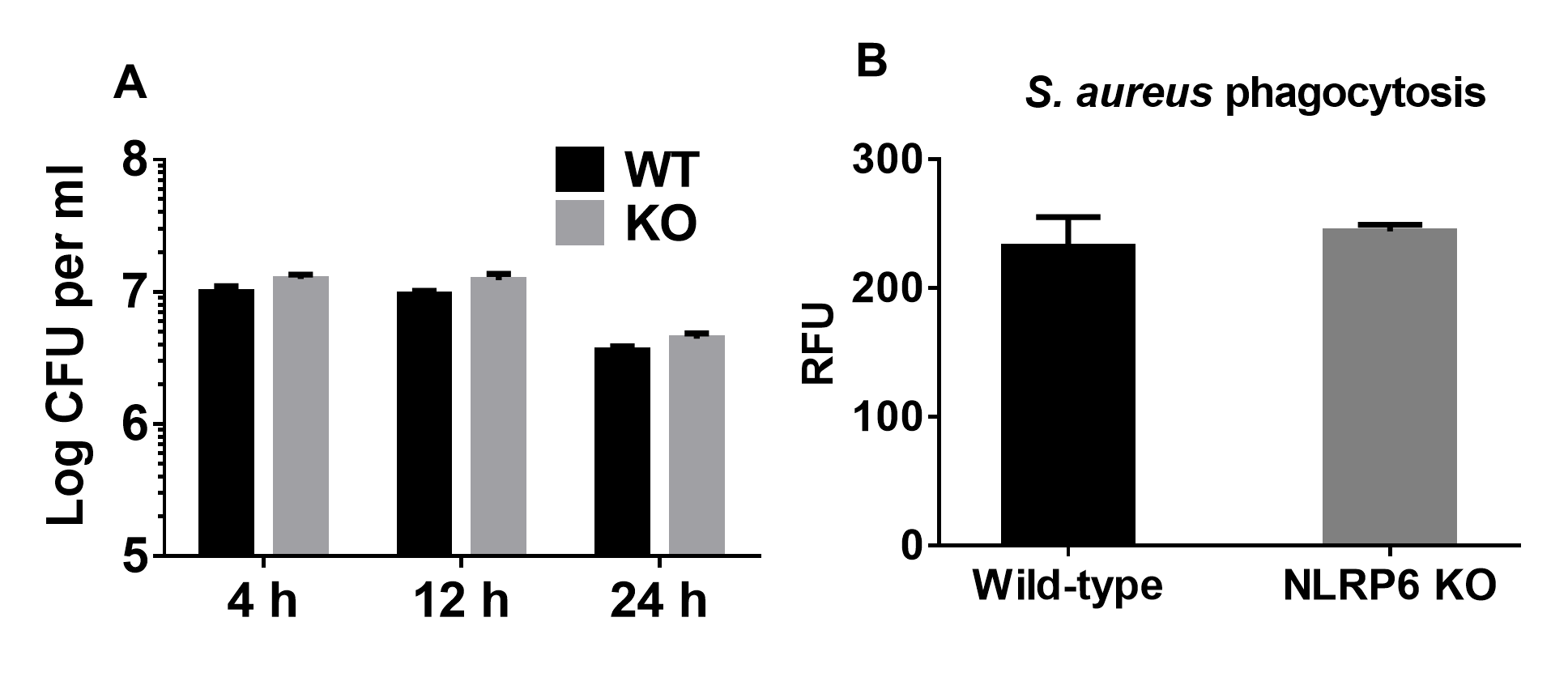
**S2 Fig: The role of NLRP6 in bacterial killing in Bone marrow-derived macrophages (BMDM). (A)** BMDM from WT and KO mice were isolated and infected with MRSA (MOI: 10). Killing capacity was compared at indicated time points as described in methods section. **(B)** Rate of phagocytosis by bone marrow-derived neutrophils (BMDN). BMDN from WT and KO mice were isolated and rate of phagocytosis was measured after one hour using pHrodo red *S. aureus* bio-particles. Each figure is a representative figure of 3 independent experiments.
