# Supplementary material for: NLRP6 negatively regulates pulmonary host defense in Gram-positive bacterial infection through modulating neutrophil recruitment and function
Source: PLoS Pathog. 2018 Sep 24;14(9):e1007308. doi: 10.1371/journal.ppat.1007308 (PMC6171945; doi:10.1371/journal.ppat.1007308)

**S3 Fig: Cellular source of IFN-γ in pulmonary MRSA infection.** WT and KO mice (N=9-11/group) were infected intra-tracheally with MRSA (5X10^7^ CFU/mouse). After 24 hours of infection, mice were euthanized to collect lungs. Single cell suspensions obtained from lungs were stimulated with PMA/ionomycin along with Brefeldin A for 4 hours and then stained intracellularly for IFN-γ. **(A)** Gating strategy to obtain cell positive for both γδ T cells and IFN-γ. **(B)** IFN-γ positive CD8^+^T cells. **(C)** Quantification of **A**. **(D)** Quantification of **B**. **(E)** NK cells and CD4 T cells **(F)** were isolated from WT and KO mice and pre-treated them with MAPK inhibitor (10µM) prior to infection with *S. aureus*. Cells were then stained intra-cellularly to detect IFN-γ positive NK and CD4 T cells. Percentage of IFN-γ positive cells are shown. Each figure is a representative of 3 independent experiments.


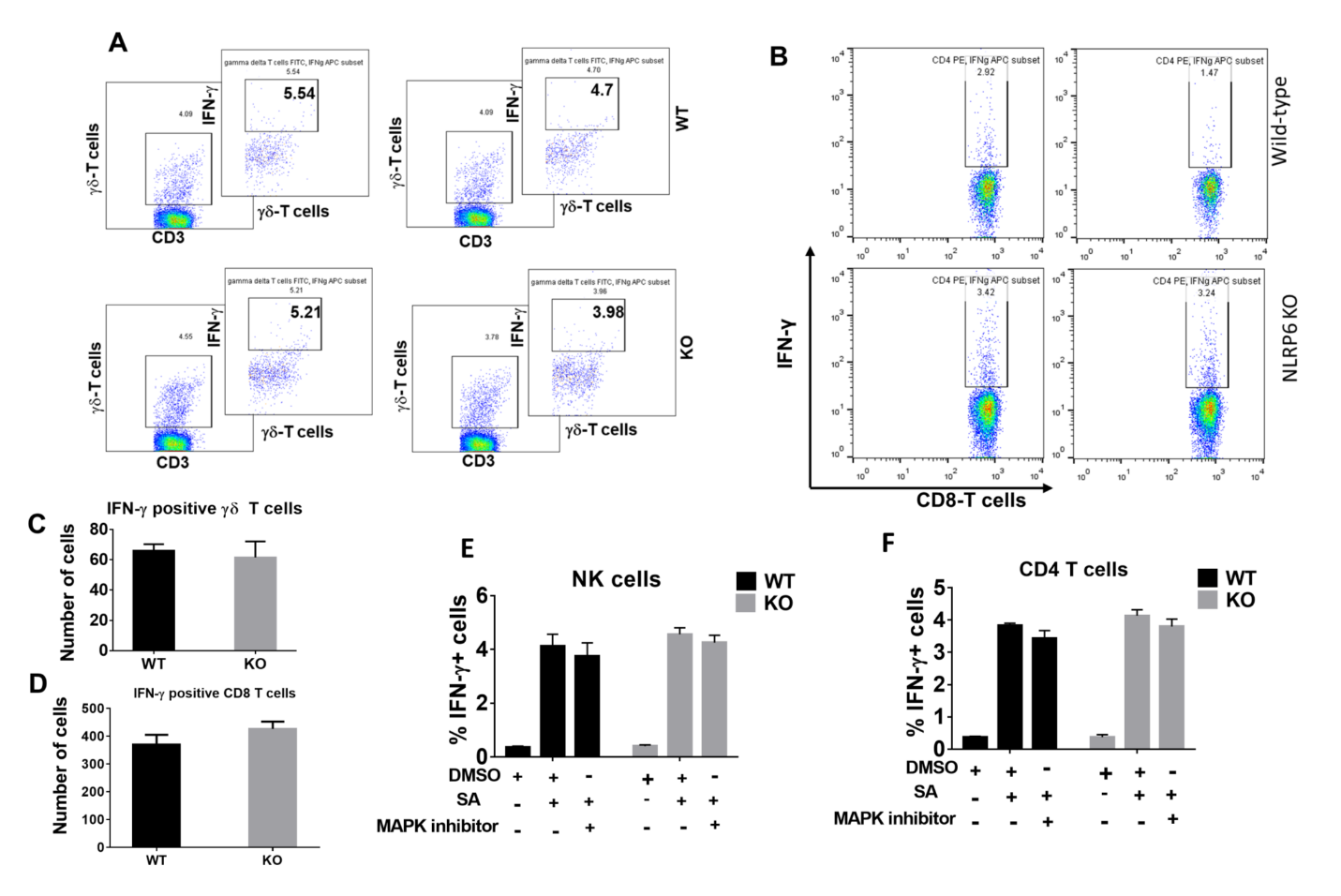

Supplement: S3 Fig — WT and KO mice (N = 9-11/group) were infected intra-tracheally with MRSA (5X107 CFU/mouse). After 24 hours of infection, mice were euthanized to collect lungs. Single cell suspensions obtained from lungs were stimulated with PMA/ionomycin along with Brefeldin A for 4 hours and then stained intracellularly for IFN-γ. (A) Gating strategy to obtain cell positive for both γδT cells and IFN-γ. (B) IFN-γ positive CD8+T cells. (C) Quantification of A. (D) Quantification of B. (E) NK cells and CD4 T cells (F) were isolated from WT and KO mice and pre-treated them with MAPK inhibitor (10μM) prior to infection with S. aureus. Cells were then stained intra-cellularly to detect IFN-γ positive NK and CD4 T cells. Percentage of IFN-γ positive cells are shown. Each figure is a representative of 3 independent experiments. (DOCX) [file ppat.1007308.s003.docx]
