# Supplementary material for: NLRP6 negatively regulates pulmonary host defense in Gram-positive bacterial infection through modulating neutrophil recruitment and function
Source: PLoS Pathog. 2018 Sep 24;14(9):e1007308. doi: 10.1371/journal.ppat.1007308 (PMC6171945; doi:10.1371/journal.ppat.1007308)
Supplement: S4 Fig — BMDM from WT and KO mice were infected with MRSA (MOI:50) for 8 hours and stained with antibodies against caspase-1, gasdermin-D, RIP3, and phopho-MLKL. Percentage of cells positive for caspase-1 (A), gasdermin-D (B), and RIP3 and p-MLKL (C) were calculated and represented in the graph. The graph is the representative of 3 independent experiments. *, p<0.05, **, p<0.01, ***, p<0.001. (DOCX) [file ppat.1007308.s004.docx]

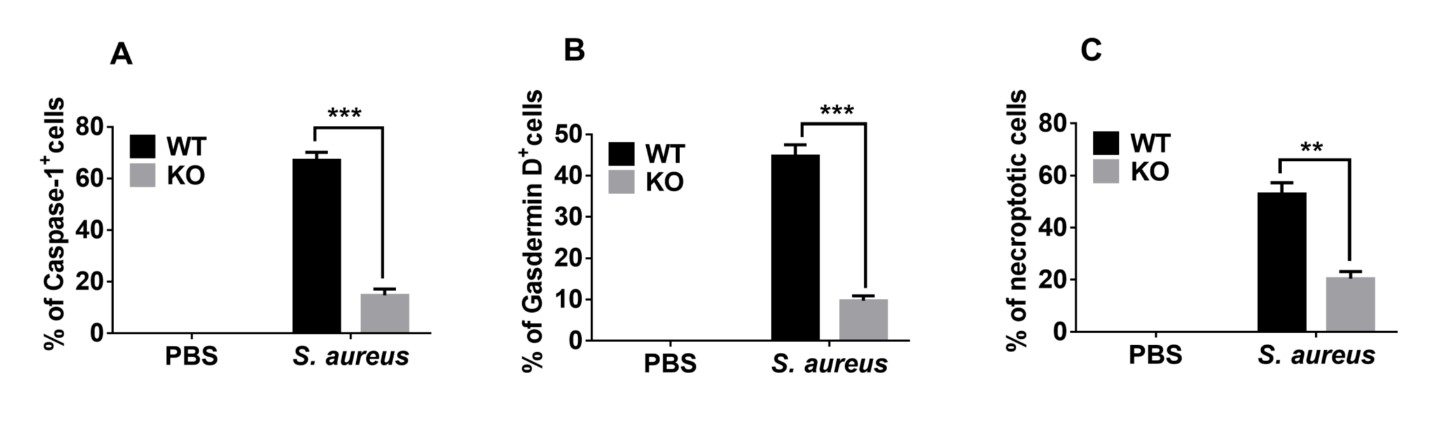
**S4 Fig:** **Role of** **NLRP6 in necroptosis during MRSA infection in bone marrow-derived macrophages (BMDM)**. BMDM from WT and KO mice were infected with MRSA (MOI:50) for 8 hours and stained with antibodies against caspase-1, gasdermin-D, RIP3, and phospho-MLKL. Percentage of cells positive for caspase-1 (A), gasdermin-D (B), and RIP3 and p-MLKL (C) were calculated and represented in the graph. The graph is the representative of 3 independent experiments. *, p<0.05, **, p<0.01, ***, p<0.001.
