# Supplementary material for: NLRP6 negatively regulates pulmonary host defense in Gram-positive bacterial infection through modulating neutrophil recruitment and function
Source: PLoS Pathog. 2018 Sep 24;14(9):e1007308. doi: 10.1371/journal.ppat.1007308 (PMC6171945; doi:10.1371/journal.ppat.1007308)

**S5 Fig: Effect of blocking of necroptosis in WT and KO mice during MRSA infection.** WT and KO mice (N=6-8/group) were treated with either Nec-1s or vehicle control 18 hours before and at the time of infection with MRSA. Twenty-four hours post-infection, mice were euthanized to measure total protein leakage **(A)** and extent of cell death **(B)** in the BALF. Each figure is a representative figure of at least 3 independent experiments. Nec-1s: Necrostatin-1s *, p<0.05.


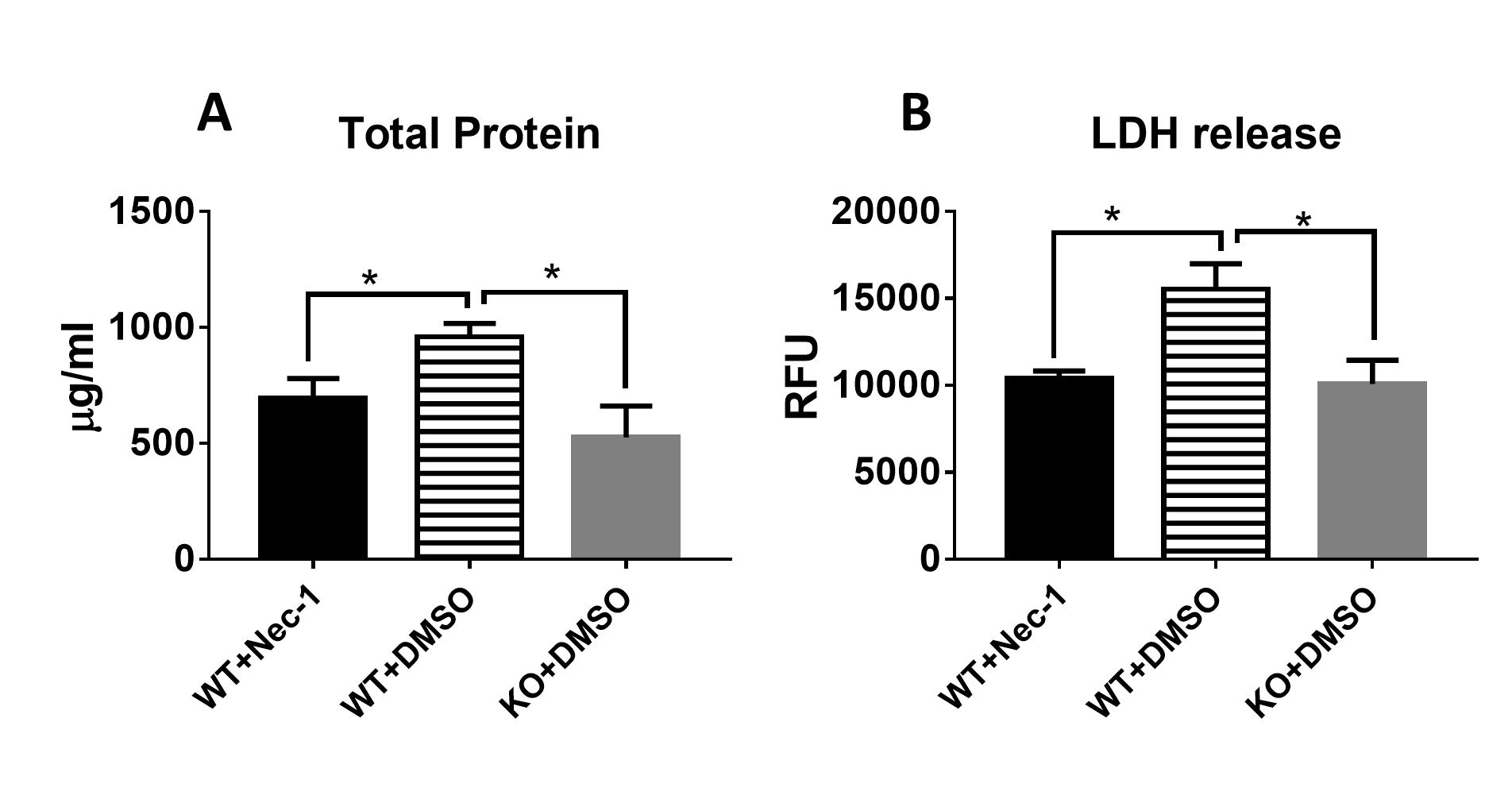

Supplement: S5 Fig — WT and KO mice (N = 6-8/group) were treated with either Nec-1s or vehicle control 18 hours before and at the time of infection with MRSA. Twenty-four hours post-infection, mice were euthanized to measure total protein leakage (A) and extent of cell death (B) in the BALF. Each figure is a representative figure of at least 3 independent experiments. Nec-1s: Necrostatin-1s *, p<0.05. (DOCX) [file ppat.1007308.s005.docx]
