# Supplementary material for: NLRP6 negatively regulates pulmonary host defense in Gram-positive bacterial infection through modulating neutrophil recruitment and function
Source: PLoS Pathog. 2018 Sep 24;14(9):e1007308. doi: 10.1371/journal.ppat.1007308 (PMC6171945; doi:10.1371/journal.ppat.1007308)

**S6 Fig:** **A scheme demonstrating how NLRP6 signaling induces mortality following pulmonary MRSA infection**. Upon infection in the lungs, MRSA upregulates and activates NLRP6 inflammasome **(1)**. The activated NLRP6 inflammasome dampens IFN-γ production by NK-cells **(2)**. Since IFN-γ is important for NADPH oxidase activity, reduction of IFN-γ secretion leads to reduced activity of NADPH oxidase **(3)** and ultimately reduced ROS production **(4)** ultimately compromising the function of neutrophils **(5)**. Reduced ROS production by neutrophils leads to reduced bacterial clearance **(6)**. Upon activation, NLRP6 increases the activity of caspase-1 and gasdermin-D **(7)** thereby triggering pyroptosis **(8)**. Similarly, NLRP6 triggers necroptosis by upregulating RIP-3 and p-MLKL in the lungs **(9&10)**. Both of these inflammatory cell death mechanisms **(11)** lead to exaggerated inflammation and loss of immune cells **(12)**. Ultimately, there will be less bacterial clearance **(5)** and increased mortality **(13)**.


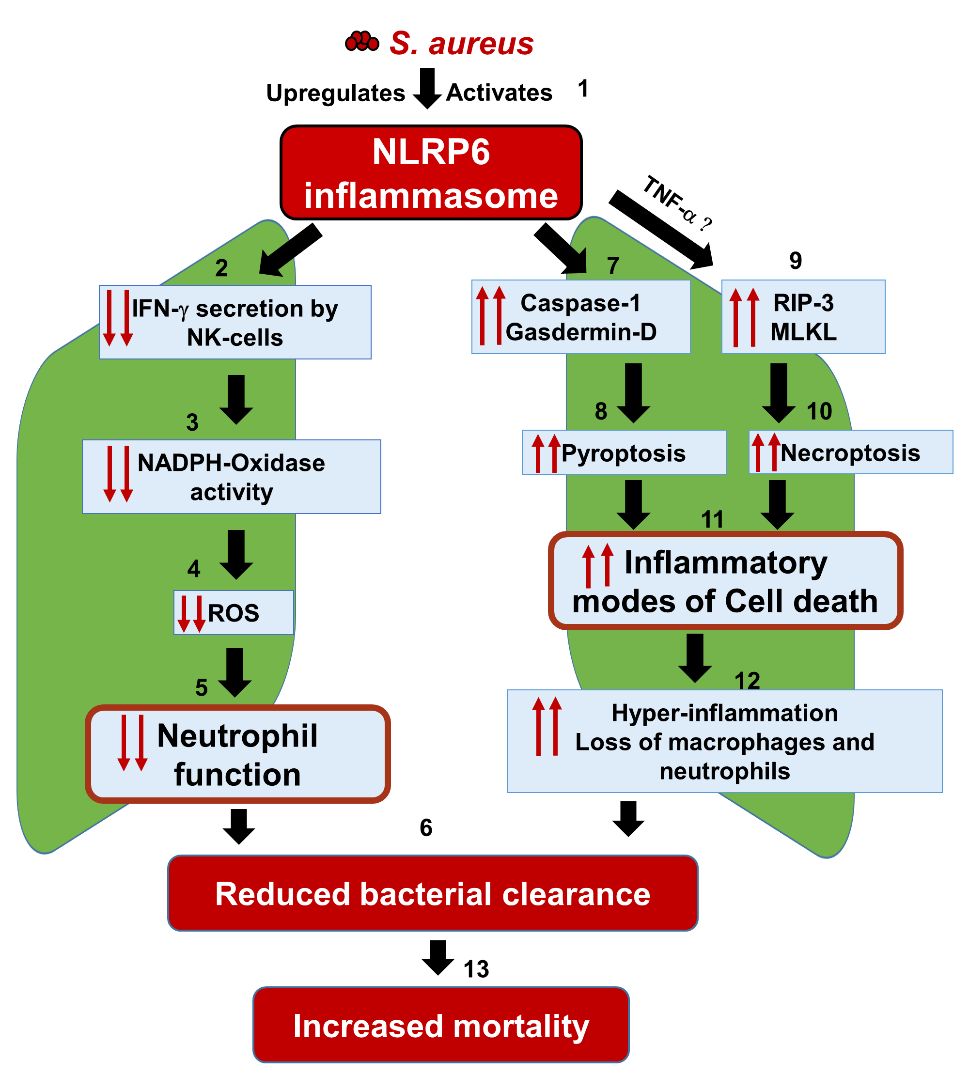

Supplement: S6 Fig — Upon infection in the lungs, MRSA upregulates and activates NLRP6 inflammasome (1). The activated NLRP6 inflammasome dampens IFN-γ production by NK-cells (2). Since IFN-γ is important for NADPH oxidase activity, reduction of IFN-γ secretion leads to reduced activity of NADPH oxidase (3) and ultimately reduced ROS production (4) ultimately compromising the function of neutrophils (5). Reduced ROS production by neutrophils leads to reduced bacterial clearance (6). Upon activation, NLRP6 increases the activity of caspase-1 and gasdermin-D (7) thereby triggering pyroptosis (8). Similarly, NLRP6 triggers necroptosis by upregulating RIP-3 and p-MLKL in the lungs (9&10). Both of these inflammatory cell death mechanisms (11) lead to exaggerated inflammation and loss of immune cells (12). Ultimately, there will be less bacterial clearance (5) and increased mortality (13). (DOCX) [file ppat.1007308.s006.docx]
